# *Ricinus communis* Intoxications in Human and Veterinary Medicine-A Summary of Real Cases

**DOI:** 10.3390/toxins3101332

**Published:** 2011-10-24

**Authors:** Sylvia Worbs, Kernt Köhler, Diana Pauly, Marc-André Avondet, Martin Schaer, Martin B. Dorner, Brigitte G. Dorner

**Affiliations:** 1 Centre for Biological Security, Microbial Toxins (ZBS3), Robert Koch-Institut, Nordufer 20, Berlin 13353, Germany; Email: worbss@rki.de (S.W.); paulyd@rki.de (D.P.); dornerm@rki.de (M.B.D.); 2 Institute of Veterinary Pathology, Justus Liebig University Giessen, Frankfurter Street 96, Giessen 35392, Germany; Email: Kernt.Koehler@vetmed.uni-giessen.de; 3 Biology and Chemistry Section, Federal Department of Defence, Civil Protection and Sports DDPS SPIEZ LABORATORY, Austrasse 1, Spiez CH-3700, Switzerland; Email: marc.avondet@babs.admin.ch (M.-A.A.); Martin.Schaer@babs.admin.ch (M.S.)

**Keywords:** ricin, poisoning, animal intoxication, human intoxication, fertilizer

## Abstract

Accidental and intended *Ricinus communis* intoxications in humans and animals have been known for centuries but the causative agent remained elusive until 1888 when Stillmark attributed the toxicity to the lectin ricin. *Ricinus communis* is grown worldwide on an industrial scale for the production of castor oil. As by-product in castor oil production ricin is mass produced above 1 million tons per year. On the basis of its availability, toxicity, ease of preparation and the current lack of medical countermeasures, ricin has gained attention as potential biological warfare agent. The seeds also contain the less toxic, but highly homologous *Ricinus communis* agglutinin and the alkaloid ricinine, and especially the latter can be used to track intoxications. After oil extraction and detoxification, the defatted press cake is used as organic fertilizer and as low-value feed. In this context there have been sporadic reports from different countries describing animal intoxications after uptake of obviously insufficiently detoxified fertilizer. Observations in Germany over several years, however, have led us to speculate that the detoxification process is not always performed thoroughly and controlled, calling for international regulations which clearly state a ricin threshold in fertilizer. In this review we summarize knowledge on intended and unintended poisoning with ricin or castor seeds both in humans and animals, with a particular emphasis on intoxications due to improperly detoxified castor bean meal and forensic analysis.

## 1. Introduction

**Figure 1:**
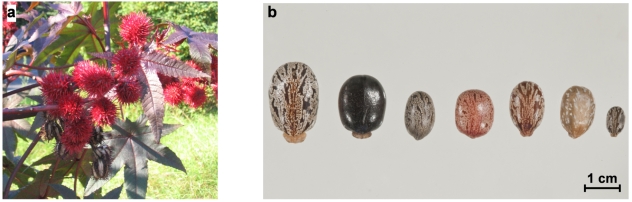
*Ricinus communis* (**a**) The castor oil plant *Ricinus communis* with characteristic seed pods; (**b**) Seeds of *Ricinus communis* varieties showing the diversity of different *R. communis* cultivars. From left to right: *R. c. zanzibariensis*, *R. c. zanzibariensis*, *R. c. green giant*, *R. c. zanzibariensis*, *R. c. carmencita*, *R. c. india*, *R. c. tanzania*.

The castor oil plant *Ricinus communis*, also known as *Palma(e) Christi* or wonder tree, is a perennial scrub of the spurge family *Euphorbiaceae* (Figure 1a). *Ricinus communis* probably originates from Africa and was used in ancient Egypt and by the Romans and Greeks [1,2,3]. Nowadays the plant grows wild in many tropical and subtropical regions and is found as an ornamental plant virtually all around the world. Historically, the plant, the seeds and in particular the oil have been used for a variety of medical purposes, for example, as a laxative or for treatment of infection and inflammation [1]. Castor seeds are a rich source of oil which can be extracted by milling, boiling, pressing or solvent extraction. Apart from medical applications, the oil has long been used as an inexpensive fuel for oil lamps. Because of its high proportion of the fatty acid ricinoleic acid, today it is a valued industrial raw material for lubricants, paints, coats, cosmetic products and many more [4,5]. Interestingly, in western Africa alkaline-fermented castor seeds are part of the flavoring soup condiment *ogiri* [6,7]. Recently, *Ricinus communis* and other *Euphorbiaceae* like *Jatropha curcas* gained interest as non-food oil seed trees for biofuel/biodiesel production [8,9]. Today, about 1 Mt of castor beans are harvested annually for castor oil production, with India, China and Brazil being major producers [5]. The plant, and in particular the seeds after oil extraction, are a rich source of protein and have been used to supplement feed, following detoxification, intended for, e.g., sheep, cattle, chicken and fish rations [10,11,12,13,14,15,16,17]. In fact, the major application of castor seed residual matter is as fertilizer or organic manure [18,19,20,21]. Generally, the use of castor bean meal, press cake or other residues of the castor oil production as a protein source for feed or fertilizer is limited by the toxicity of the seeds, mainly caused by the highly toxic protein ricin and the less toxic alkaloid ricinine. Ricin is a water-soluble protein and is thereby not extracted into the castor oil, therefore industrial grade castor oil has been found to be safe [22]. Various methods including physical, chemical and biological treatment have been employed to detoxify the residues of industrial castor oil production to be used for feeding or other purposes [18,19,23,24,25,26,27]. To surmount the problem of toxicity, researchers have attempted to obtain a non-toxic castor cultivar, so far with limited success [28,29,30,31].

## 2. Ricin, a Toxic Lectin from *Ricinus communis*

While the toxicity of *Ricinus communis* has been known for centuries, it was only through the seminal work of Kobert’s group at the University of Dorpat (now University of Tartu, Estonia) on plant toxalbumins that the toxic principle of *Ricinus communis* was attributed to a protein termed ricin [32,33]. Today we know that ricin is contained within the seeds at a percentage of up to 5% [34]. Nearly a century later the toxic principle of ricin was elucidated by Endo and co-workers when they identified ricin and other plant toxalbumins as RNA *N*-glycosidases (EC 3.2.2.22 within the enzyme nomenclature of the International Union of Biochemistry and Molecular Biology), also known as ribosome-inactivating proteins (RIPs) [35,36]. For biosynthesis of ricin in *Ricinus communis,* please refer to the excellent review by Lord and Spooner in this special issue of Toxins [37]. Ricin, a prototype AB toxin, consists of a catalytically active A-chain (RNA *N*-glycosidase) and a sugar-binding B-chain (lectin) linked via a disulfide bond [38]. Cell binding occurs through the B-chain and involves different oligosaccharide residues on the cell surface. Several oligosaccharide residues, including *N*-acetylglucosamine and galactose residues on glycolipids and glycoproteins, are known receptors for the lectin subunit, and these oligosaccharides show a broad and abundant presence on mammalian cells [39,40,41]. In fact, various oligosaccharides have been used for purification of ricin by affinity chromatography [42,43,44,45]. The understanding of ricin (RCA60) was complicated by the presence of a homologous protein, later identified as *Ricinus communis* agglutinin (RCA120), a much less toxic dimeric protein with high sequence identity to ricin. The co-existence of two highly similar proteins, one a potent cytotoxin (RCA60), the other an effective haemagglutinin (RCA120), came to light by improved separation methods and by molecular identification of the two different genes [41,46]. Later, an isoform of ricin named ricin E (while the original ricin is now termed ricin D) was discovered both on protein and on DNA levels to contain a hybrid B-chain of ricin and *R. communis* agglutinin, adding further complexity to the issue [47,48,49]. Whereas ricin is a monomeric AB toxin of about 60 kDa formed by a covalently linked A- (~32 kDa) and B-subunit (~34 kDa), *R. communis* agglutinin is a ~120 kDa homodimer of two A- (~32 kDa) and B-subunits (~36 kDa) [50]; in one publication, a disulphide bond between the two A-chains of RCA120 has been shown by X-ray crystallography [51]. On amino acid levels, both the A- and the B-chains of RCA60 and RCA120 show a high degree of homology of 93% and 84%, respectively [46], reflecting similar but not identical structures and biochemical properties [52,53]. The corresponding B-chains of RCA60 and RCA120 are not as highly conserved as the A-chains, but still bind to identical oligosaccharides like β-1,4-linked galactose residues; additionally, ricin shows selective binding to *N*-acetylglucosamine oligosaccharides [39,41]. Both A-chains isolated inhibit ribosome activity in a cell-free system, however, the A-chain of RCA120 to a lesser extent (5 to 14-fold; [54,55]). The difference in toxicity between ricin and agglutinin is much more pronounced, with ricin being about 100-2000 times more toxic than agglutinin, depending on the experimental system used [41,43,53,56]. This might be due to the slightly different binding repertoire of the B-chains [39,57] and, additionally, differences in the haemagglutination activity of ricin and agglutinin. *R. communis* agglutinin, on the other hand, shows a much more profound haemagglutination activity than ricin [41,43], leading to the speculation that a high proportion of agglutinin might bind to serum glycoproteins or erythrocytes and might not be available for its toxic action [53]. 

The journey of ricin from the cellular surface to the ribosome has been the focus of recent research, highlighting common uptake and transport mechanisms also described for other proteins (for review see this special issue of Toxins, Lord and Spooner 2011 and [58,59,60,61]). Ricin, with its lectin subunit (B-chain), binds to oligosaccharide residues on the cell surface and undergoes endocytosis via clathrin-dependent and -independent mechanisms that are somewhat dependent on the cell type and polarisation status studied [62,63,64,65,66,67]. Internalized ricin reaches the early endosomal compartment from where the majority is recycled or undergoes degradation in the lysosomes, whereas only a minor fraction reaches the trans-Golgi network [64,68,69,70,71,72,73]. Once in the Golgi, ricin is transported retrogradely to the endoplasmic reticulum (ER) by yet unexplored pathways [74,75,76,77,78]. Until ricin has reached the ER it still consists of a heterodimer of the A- and B-subunit; within the ER it is reduced by disulfide isomerase and separates into the two chains [79,80]. In the ER the ricin A-chain subverts the so-called ER-associated degradation process, by which misfolded proteins are eliminated, and is transported into the cytosol [81,82,83,84,85,86,87,88]. Finally, after retrotranslocation into the cytosol, the A-chain binds to the ribosomal stalk of the ribosome [89]. At the ribosome it removes an adenine from the so-called sarcin-loop of the 28S rRNA, thereby preventing binding of elongation factors and further protein synthesis [89,90,91]. Apart from this major interruption of cellular function, ricin is also capable of inducing apoptosis by yet not fully understood mechanisms [92,93,94,95,96,97]. 

The endogenous function of ricin within the plant remains elusive; based on the cytotoxic activity it is speculated that it might function in the defense against all sorts of plant-eating or -damaging organisms [98,99,100,101].

## 3. Ricin, a Dual-Use Substance

On the one hand, the ricin-producing plant is of economic interest for the production of castor oil and the numerous industrial, medical and cosmetic products derived thereof. The oil contains high levels of the unusual fatty acid ricinoleic acid that is valued for its unique chemical properties. Furthermore, with respect to medical applications, the ability of the A-subunit to induce cell death has been exploited for the development of immunotoxins. Immunotoxins combine the toxic principle of a toxin with the exquisite binding specificity of antibodies in one chimeric molecule [102]. Ricin A-chain was one of the first examples of a toxin coupled to monoclonal antibodies against cell surface proteins and was used experimentally for the treatment of various cancers [103,104,105,106]. However, unexpected side effects like the so-called vascular leak syndrome hampered the efforts [107,108,109], but progress has been made recently including phase I or III clinical trials, respectively [106,110].

On the other hand, ricin has attracted dangerous interest as it has a history of military, criminal and terroristic use. The toxin has been explored for potential military use by different nations. It was included in different weapons programs during World War II (codename: compound W), and weaponised material was later produced until the 1980s [111,112,113,114]. Based on its history, ricin is a prohibited substance both under the Chemical Weapons Convention (CWC, schedule 1 compound) and the Biological Weapons Convention (BWC) and its possession or purification is strictly regulated and controlled by the Organization for the Prohibition of Chemical Weapons (OPCW). The relative ease in preparing a crude extract and the world-wide availability of the plant has also made ricin a potential agent of bioterrorism. It is therefore listed as category B agent of potential bioterrorism risk by the Centers for Disease Control and Prevention [115,116]. Ricin has gained notoriety as the most likely agent used in the assassination of the Bulgarian dissident Georgi Markov in London in 1978 and the attempted murder of Vladimir Kostov in Paris (Table 1; [117]). In the past, the focus fell on the toxin for criminal use and various attempted acts of bioterrorism. To provide a few examples, ricin was found in threat letters to members of the US Senate and the White House (in 2003/2004); *Ricinus communis* seeds and means for the preparation of ricin have been discovered during a raid against terrorists in London in 2002. In a number of cases worldwide, the production and possession of ricin has been well documented. These aspects of ricin are reviewed by Griffiths in this special issue of Toxins.

## 4. Toxicity of Ricin and *R. communis,* Agglutinin

When assessing the numerous reports on intoxications with ricin, *R. communis* seeds or *R. communis*-containing feed and fertilizer, some general aspects have to be considered. The term ricin in any toxicological publication suggests a degree of homogeneity or a lack of variability that might be expected for pure chemicals. Proteins, however, are usually purified and extracted from living sources and show a more or less endogenous variability which has to be kept in mind when comparing toxicities given in different publications. For *R. communis,* a large number of different cultivars are known, and the high variability of the cultivars can be nicely demonstrated on the plant and also on the seeds which are phenotypically quite diverse. As shown in Figure 1b, *R. communis zanzibariensis* is particular among the *R. communis* cultivars as it comes in different seed shapes and colors, and it can also be clearly distinguished from other cultivars based on its biochemical characteristics [118,119]. Small and large seeds of different cultivars have been reported to contain different levels of ricin D and ricin E, respectively [47,118,120]. As mentioned above, ricin is not the only toxic protein in the seeds, it shares a high degree of homology with *R. communis* agglutinin. Recently, sophisticated sequence analysis methods have revealed that ricin and *R. communis* agglutinin are not single-copy genes. Rather they are members of a ricin gene family encoding seven full-length ricin or ricin-like proteins and several potential shorter gene products of unknown expression and function, reflecting a much greater variability than previously anticipated [121,122]. The full-length proteins of the ricin gene family have been shown to inhibit protein synthesis similar to ricin itself [121]. Additional heterogeneity of ricin is based on different glycosylation patterns [118], and variable toxicities of ricin isoforms have been correlated with different glycosylation levels [123,124].

Based on the variability described, it could be retrospectively assumed that the toxicity of ricin has mostly been determined with toxin preparations containing a mixture of differently glycosylated ricin isoforms (which might or might not contain *R. communis* agglutinin to a variable degree, especially in the older literature when chromatographic separation techniques were not as advanced as today). Toxicity data might also depend on the application of different purification protocols, including acid precipitation (may influence [re-]folding), elution with sugars (may affect B-chain binding) or salt conditions, all resulting not only in different purities but also different functional activities [125]. Furthermore, a certain degree of variability in toxicity data is linked to the experimental system used, e.g., the animal species or strain used and the cell culture or *in vitro* assay used [126,127,128]. Considering all these different points, the following numbers are the best estimates to summarize a great deal of experimental work done in different laboratories. Ricin acts in a time- and concentration-dependent manner [56,125]. Notably, there is a time delay of about 10 h before death occurs even with very high doses applied [125]. By intravenous injection of ricin into mice, the dose that produces death in 50% of animals (LD_50_) was found to lie between 2-8 µg/kg body weight [41,125,128,129,130,131,132]. In rats 0.35-0.5 µg/kg, guinea pigs 0.4-0.5 µg/kg, rabbits 0.03-0.06 µg/kg and dogs 1.65-1.75 µg/kg were reported [131]. Somewhat more divergent amounts between 2.4 and 36 µg/kg were needed to produce death in 50% of mice after intraperitoneal injection [56,128,132,133,134]. The inhalational toxicity (in estimated LD_50_) was reported to be between 2.8 and 12.5 µg/kg in different mouse strains [127,135]. Using the same application route, the LD_50_ for two different *R. communis* cultivars in rats has been reported to be between 3.7 µg/kg and 9.8 µg/kg [136,137,138]. It has to be considered that calculation of effective doses in inhalational challenging experiments is more complicated than that for injection, since the effective delivery into the deep lungs depends-among other things-on the particle size, the solvent used and the technical specifications of the aerosol chamber [135,137,139]. In non-human primates the LD_50_ after inhalational application was found to be 5.8 µg/kg for African green monkeys and 15 µg/kg for rhesus monkeys [127]. The least toxic route is oral uptake or intra-gastric delivery and is about 1000 times less toxic than parenteral injection or inhalation. For mice 21.5 mg/kg and 30 mg/kg were reported [132,140,141]. Although different values for oral LD_50_ in rats are cited in the secondary literature, clear data within the accessible primary literature are scarce; the oral LD_50_ in rats was estimated to be up to 20-30 mg/kg ([140,142]; this point is relevant since certain national regulations for *R. communis*-derived products rely on oral LD_50_ values in rats; see below). With respect to humans, the median lethal oral dose for ricin has been estimated to be 1-20 mg/kg of body weight on the basis of real cases reporting castor bean poisoning [111]. Data on the *in vivo* toxicity of purified RCA120 indicates that the protein is about 1000 times less toxic than ricin in mice after intraperitoneal injection, and an LD_50_ of ~8 mg/kg was given [41]. Others reported slightly lower LD_50_ values of 1.36 and 1.40 mg/kg after intravenous injection [56,134].

## 5. Ricinine

Apart from the highly toxic ricin and the less toxic *R. communis* agglutinin the plant contains another toxic compound, the low molecular weight alkaloid ricinine (MW = 164.2 g/mol). Ricinine or 3-cyano-4-methoxy-*N*-methyl-2-pyridone (CAS 524-40-3) belongs to the group of piperidine alkaloids. It was first discovered and named by Tuson in the seeds of *Ricinus communis* while searching for its medically active compounds even before ricin was known [143]. Subsequently, its chemical structure was identified [144,145,146,147] and its biosynthesis and metabolism was studied [148,149]. Ricinine can be found in all parts of the plant and it is a quite strong insecticide. The castor seeds contain approximately 0.2% of the alkaloid. In experimental mouse models ricinine causes hyperactivity, seizure and subsequent death due to respiratory arrest. LD_50_ values for ricinine were 340 mg/kg for intraperitoneal and 3 g/kg for oral incorporation [150]. Therefore, in comparison to ricin, ricinine is significantly less toxic. However, much smaller doses (20 mg/kg) are sufficient to induce CNS effects like seizures in mice [151,152]. Unlike ricin, ricinine cannot be inactivated by conventional heat treatment because of its high temperature resistance (melting point ~200 °C). Therefore, only after elimination of ricinine by solvent extraction is the residue from castor oil production suitable for animal feeding.

In summary, *Ricinus communis* contains a complex cocktail of toxic substances including the type II RIP ricin, the haemagglutinin RCA120 and the alkaloid ricinine. Furthermore, other compounds like fatty acids, flavonoids and saponins have been found to exhibit deleterious effects on bacteria, virus, fungi, invertebrates and higher animals, seemingly giving the plant some sort of protection in a hostile environment [153,154,155,156,157]. Furthermore, allergenic reactions against *Ricinus communis*, in particular the seed dust, were realized [158,159,160]. Low molecular proteins, 2S albumins, have been identified as the main allergenic compounds [161,162,163,164]. Experimental intoxication studies underline the major contribution of ricin compared to other hazardous compounds found in the seeds [132].

## 6. Ricin Intoxications in Humans

When reviewing case reports of ricin intoxications in humans, “effective” ricin doses that have been incorporated can only be estimated according to variations in the size, weight and moisture content of the seeds; cultivar, region, season and period of plant growth at the time of uptake as well as degree of mastication, stomach content, age and comorbidities which are obviously more heterogeneous compared to experimental poisoning of animals [111]. In clinical reports, the number of seeds ingested causing mild to severe symptoms, including a fatal outcome, range from uptake of only single seeds to up to 30 seeds [32,33,111]. Overall, the majority of intoxications occur accidentally and are due to incorporation of *Ricinus communis* seeds; only in some cases intended uptake of castor seed extracts has been documented in attempting suicide (Table 1). Fatalities after uptake of seeds mainly occurred in the pre-modern medicine era without effective supportive care. In those cases of attempted suicide where seed extracts were self-injected, the fatality rate seems to be higher (five out of seven injectional cases were fatal, Table 1), reflecting the higher toxicity after parenteral application. Human cases until 1900 are reviewed by Stillmark [33], while Balint, Rauber and Challoner summarize about 700 cases until 1990 [165,166,167]. Examples of more recent cases will be given below. Most often accidental poisoning occurs by unaware children who are attracted by the appearance of the seeds [168]; some cases describe the uptake of seeds by adults out of curiosity or because the seeds are mistaken for nuts (Table 1). 

Generally, independent of the uptake route (oral or parenteral injection) the symptoms induced by ricin were quite similar, and the severity of symptoms increases with the amount of toxin incorporated. Symptoms arose after 3 to 20 h after ingestion or injection. Physical symptoms were abdominal pain, emesis, diarrhea with or without blood, muscular pain, cramps in the limbs, circulatory collapse, dyspnoea and dehydration. Muscular pain and circulatory collapse were more commonly observed with injected ricin, as well as pain at the injection site. Biochemical analyses often revealed increase in white blood cells, blood urea nitrogen (BUN), aspartate aminotransferase (AST) and alanine aminotransferase (ALT), indicating dysfunction of liver and kidneys. Autopsy in fatal cases showed haemorrhagic necrosis in intestines and heart and oedema in lungs.

A comprehensive review from a Sri Lankan hospital records local child poisoning cases between 1984 and 2001, reporting 46 cases of accidental *Ricinus communis* intoxications (and further cases caused by intoxication with *Abrus precatorius*, *Jatropha curcas*, *Manihot utilissima*, and others), all of them not fatal; all patients experienced vomiting and some dehydration and abdominal pain [169]. Other areas where *Ricinus communis* is endemic or grown on an industrial scale also report a high number of accidental intoxications in children. From India, 57 non-fatal cases between 1962 and 1965 were reported [170]. In 1980 in the USA, a boy ingested up to four *Ricinus communis* seeds of an ornamental necklace. His mother brought him to the emergency clinic where emesis was induced, followed by charcoal treatment and cathartics. He was able to leave the hospital 72 h later [171]. The publication highlights the danger linked to the ornamental use of decorative, but toxic plant seeds. However, also in the last decade, adults including the elderly have been involved in ricin intoxications. In Malta, an elderly man was admitted to a clinic with persistent vomiting and watery diarrhea after he had eaten 10 seeds, later identified as seeds from *Ricinus communis*; he was dehydrated, tachycardic and hypotensive. Under supportive management (fluids) he fully recovered and left the hospital 7 days later [172]. In a case in Australia in 1995 a young adult ingested 10-15 *Ricinus communis* seeds out of curiosity and presented at the emergency department with persistent vomiting and abdominal pain; after successful treatment (fluids, charcoal, emetics) he was able to leave the hospital on the third day [173]. In a case in Great Britain in 1992, a chemist injected himself with a watery extract of a single seed out of curiosity, reportedly not in a suicidal attempt (containing about 150 mg ricin based on the analysis of the remaining extract). He developed severe headache and rigors, liver damage and pyrexia were observed for 8 days, but he fully recovered after 10 days [174].

A very recent review on the American Association of Poison Control Centers reports 45 fatalities out of more than 2 million plant poisonings between 1983 and 2009, of these, only one fatal case was attributed to *Ricinus communis*, while the majority (16 deaths) was caused by *Datura* and *Cicuta* species [175]. A review by the Swiss Toxicology Information Centre mentioned 130 serious cases including five fatal plant poisonings between 1966 and 1994, among them three non-fatal cases related to *Ricinus communis* [176]. These reviews of local plant poisonings support the opinion that intoxications with *Ricinus communis* usually do not belong to the most common or serious poisonings occurring accidentally in humans.

The seeds of *Ricinus communis* have a long history as medical remedy; it is therefore not surprising to find cases linked with adverse reaction to them: A Korean woman who had eaten five castor seeds in order to treat constipation was admitted to hospital with severe nausea, vomiting, abdominal pain and initially near hypothermia; in this case ricin was detected in urine samples, symptoms were treated (fluids, charcoal) and she was discharged after 2 days [177]. Similar cases have been reported from Brazil and Croatia [178,179]. In Japan a man bought *Ricinus communis* seeds to treat his rheumatic condition. Not realizing the seeds were meant to be used for dermal application in an ointment he swallowed about 30 of them; the next day he presented at a hospital with diarrhoea, vomiting and abdominal pain. Gastric lavage was performed and fluids and charcoal were given. Even in this severe case the patient recovered and could leave the hospital 8 days later [180]. A report from Oman describes the case of a man who ate one green seed of *Ricinus communis* as a traditional treatment against coughing. After vomiting he presented at the local hospital in a confused and disoriented, afebrile state with sluggish pupil reflex, mydriasis and high pulse rate. He was treated symptomatically and with charcoal, within two days he returned to normal [181]. A unique case occurred in 2009 in the USA: an unlicensed practitioner illicitly injected 500 mL of castor oil into a person for hip augmentation, the oil was intended to be used as silicone substitute [182]. The patient immediately developed severe symptoms including fever, tachycardia, haemolysis, thrombocytopenia, hepatitis, respiratory distress and anuric renal failure. After intensive supportive care (mechanical ventilation and haemodialysis), the patient was discharged 11 days later, requiring dialysis for an additional 1.5 months. In this unique case, ricinine was detected in the patient’s urine. The case also showed that ricinine can be found as a biomarker in refined “medicinal” castor oil preparations. The lack of CNS symptoms and seizures led to the assumption that the patient’s toxicity could be attributed to castor oil and ricinoleic acid [182].

In cases of intended uptake of ricin different reports describe suicides by injection of a self-made seed extract in Poland, Belgium and the US (Table 1). A fatal suicide took place in Poland, here a man subcutaneously injected himself with a *Ricinus communis* seed extract and was admitted 36 h later to the clinic with nausea, dizziness, pain and severe weakness. He deteriorated with haemorrhagic diathesis and multi-organ failure and died after asystolic arrest 18 h later [183]. The second case regards a chemist, who injected himself intravenously with a solution of acetone and crushed seeds. Asymptomatic at presentation at the emergency clinic, he quickly developed vomiting, bloody diarrhoea, hypotension and lost consciousness. He died during intensive care within 12 h [184]. In Belgium, a man poisoned himself by injecting (i.v. and i.m.) about 10 mL of a self-made acetone-extract of *Ricinus communis* seeds. Twenty-four hours post injection he presented at the hospital with vomiting, diarrhoea, nausea, vertigo, pain and severe dehydration, despite immediate intensive care he died after 9 h. Ricinine and acetone were detected in urine, blood and vitreous humor, while detection of ricin was technically not feasible [185]. Also in Belgium, a man had prepared an extract of the seeds and injected the extract to his wife and himself. Both presented at the emergency clinic 12 h later with fever. Treatment with tetanus vaccination, immune globulins, systemic antibiotics, corticosteroids and local wound care was initiated but both developed necrotising fasciitis. Despite extensive debridement or amputation their conditions worsened and finally resulted in death. Ricin was found in the content of the syringe and in the urine [186]. As mentioned above, compared to oral uptake, injections of *R. communis* seed extracts typically result in a much more severe clinical course but must not necessarily be fatal as a case from France illustrates. In a suicide attempt, a man with depression chewed and masticated 13 seeds and injected the product into his thigh. Necrotic tissue was excised by emergency surgery and antibiotics were given, but three further operations were necessary to remove necrotising tissue before the patient’s condition improved so that he could be discharged after 3 months [187].

Overall-among all plant poisonings reported-human cases of ricin poisoning are rare. With modern supportive care the fatality rate is low, except in suicide cases where a ricin-containing extract is injected, reflecting the higher toxicity after parenteral application.

**Figure 2:**
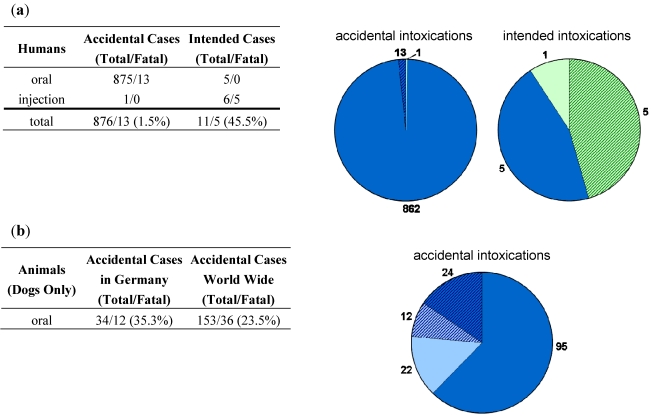
Summary of human and veterinary intoxications with ricin as displayed in detail in Table 1 and Table 2. (**a**) Human intoxications with ricin as displayed in detail in Table 1. Human cases are presented either as accidental or intended intoxications and are further sub-divided into oral and injectional intoxications. The number of cases reported and the number of fatal cases among them are given within the table (left) and as pie chart (right) with number of oral cases (blue), injectional cases (green) and the number of fatal cases highlighted (hatched); (**b**) For veterinary intoxications with ricin, details on cases occurring in dogs are summarized as shown in detail in Table 1. The table (left) and the corresponding pie chart (right) show the number of dogs poisoned accidentally in Germany (pale blue) and world-wide (blue) and the number of fatal cases (hatched). Cases mentioned by Milewski *et al.* were not considered because of lack of information on the outcome of intoxication [188].

**Table 1:** Summary of human intoxications with ricin.

Human Cases: Accidental					
	Uptake/Ingestion	Outcome	Where	Detection and Diagnosis ^1^	Ref. ^2^
101 people (different age)	ingestion and injection of varying amounts of *Ricinus communis* derivates	6 fatal	worldwide	circumstantial evidence	[33]
man (age 26)	in 1900 ingestion of unknown amount of castor seeds	recovered	UK	circumstantial evidence	[189]
juvenile (age 15)	in 1902 ingestion of 10 or 12 castor seeds	fatal	USA	circumstantial evidence	[190]
4 adults (age unknown)	in 1903 ingestion of 1, 4, 6 and 14 castor seeds, respectively	recovered	Cuba	circumstantial evidence	[190]
man (age 50)	in 1903 ingestion of 2 castor seeds	recovered	UK	circumstantial evidence	[191]
man (age unknown)	in 1920 ingestion of 5 castor seeds	fatal	USA	circumstantial evidence	[192]
2 women (age 22 and 41)	ingestion of 2.5-5 castor seeds for treatment of stomach convulsions	fatal	Hungary	circumstantial evidence	[193]
man (age unknown)	drinking of an extract made of a fistful castor seeds	fatal	Hungary	circumstantial evidence	[193]
man (age 24)	in 1934 ingestion of 15-20 castor seeds	fatal	Germany	circumstantial evidence	[194]
4 adults (age unknown)	ingestion of 1-15 castor seeds	recovered	Austria	circumstantial evidence	[195]
child (age 7)	in 1941 ingestion of 4 castor seeds	recovered	Italy	circumstantial evidence	[196]
woman (age 60)	in 1948 ingestion of 10 castor seeds for relaxant	recovered	Brazil	circumstantial evidence	[178]
2 people (age unknown)	in 1950-1952 ingestion of unknown amount of castor seeds	recovered	Italy	suspected	[197]
10 children (age 11-13)	in 1958 ingestion of 0.5-6 castor seeds	recovered	Hungary	circumstantial evidence	[198]
man (age 42)	ingestion of 10 seeds	recovered	Poland	circumstantial evidence	[199]
57 children (age 1-5, >5)	in 1962-1965 ingestion of unknown amount of castor seeds, on average 4-5	recovered	India	circumstantial evidence	[170]
443 children (age <19)	in 1964-1969 ingestion of unknown amount of castor seeds	recovered	USA	suspected	[200]
man (age 57)	in 1970 ingestion of unknown amount of castor seeds, which were thought to be scarlet runner beans	recovered	Netherlands	circumstantial evidence	[201]
4 men (age 7-18)	ingestion of 1-2 castor seeds	recovered	India	circumstantial evidence	[202]
family of 4 people (age 8-44)	in 1974 ingestion of 2-10 castor seeds	recovered	Italy	circumstantial evidence	[203]
girl (age 17)	in 1965 ingestion of 1 castor seed	recovered	UK	circumstantial evidence	[204]
child (age unknown)	In the 1970s ingestion of unknown amount of castor seeds	recovered	India	circumstantial evidence	[205]
boy (age 4)	in 1979 ingestion of 4 castor seeds from an ornamental necklace	recovered	USA	circumstantial evidence	[171]
girl (age 2)	in 1979 ingestion of at least 1 castor seed	recovered	USA	circumstantial evidence	[171]
7 children (age unknown)	in 1968-1970 ingestion of 1-10 castor seeds because of good taste	recovered	Croatia	circumstantial evidence	[179]
7 adults (age unknown)	in 1968-1970 ingestion of 1-10 castor seeds as laxative	recovered	Croatia	circumstantial evidence	[179]
2 children (ages 4 and 5)	in 1979 ingestion of unknown amount of castor seeds which were found in a canister together with walnuts	recovered	USA	circumstantial evidence	[206]
4 adults (age 19-21)	ingestion of 0.5-1 castor seed	recovered	Denmark	circumstantial evidence	[207]
10 children (age 6-8)	ingestion of 1-7 castor seeds and just contact, respectively	recovered	Spain	circumstantial evidence	[208]
2 boys (age 17)	ingestion of 8 and 3 castor seeds	recovered	Israel	circumstantial evidence	[209]
9 children (age 7 to 12)	in 1984 ingestion of 1-2 castor seeds which were taken to school	recovered	USA	circumstantial evidence	[165]
child (age 11)	ingestion of 1 castor seed during lesson in school	recovered	UK	circumstantial evidence	[210]
man (age 21)	ingestion of 12 castor seeds, which were thought to be hazelnuts	recovered	USA	circumstantial evidence	[211]
woman (age 80)	ingestion of unknown amount of shelled castor seeds out of ambiguous reasons	recovered	USA	circumstantial evidence	[211]
woman (age 52)	ingestion of 10-15 castor seeds without knowledge of its toxicity	recovered	Spain	circumstantial evidence	[212]
child (age 3)	ingestion of two or more castor seeds	recovered	USA	suspected	[166]
man (age 28)	ingestion of 4 castor seeds as treatment against constipation	recovered	USA	circumstantial evidence	[166]
man (age 39)	ingestion of 4 castor seeds, man declared, that he often eats roasted castor seeds	recovered	USA	circumstantial evidence	[166]
28 children (age < 15)	in 1986 ingestion of unknown amount of castor seeds	recovered	Sri Lanka	suspected	[213]
man (age 36)	extract of 1 castor seed was injected, against migraine or out of curiosity	recovered	UK	circumstantial evidence	[174]
3 patients (age unknown)	in 1966-1994 ingestion of unknown amount of castor seeds	recovered	Switzerland	circumstantial evidence	[176]
young adult (age unknown)	in 1995 ingestion of 10-15 castor seeds out of curiosity	recovered	Australia	circumstantial evidence	[173]
girl (age 20 months)	ingestion of 2 or more castor seeds	recovered	Canada	circumstantial evidence	[214]
5 people (age unknown)	ingestion of unknown amount of castor seeds	recovered	Tunisia	not described	[215]
man (age 70)	ingestion of 10 castor seeds	recovered	Malta	circumstantial evidence	[172]
120 people (different age)	in 1955 ingestion of varying amounts of castor seeds	1 fatal	Europe	circumstantial evidence	[216]
child (age 3)	ingestion of 5-6 castor seeds	recovered	Germany	circumstantial evidence	[216]
man (age 69)	swallowing of 30 seeds without chewing; intended use: medical treatment of rheumatism, external application was recommended	recovered	Japan	circumstantial evidence	[180]
46 children (age unknown)	in 1984-2001 ingestion of unknown amount of castor seeds	recovered	Sri Lanka	suspected	[169]
man (age 51)	ingestion of one green fruit of castor plant as treatment against cough	recovered	Oman	circumstantial evidence	[181]
woman (age unknown)	injection of 500 mL castor oil for hip augmentation by unlicensed practitioner	recovered	USA	detection of ricinine in urine	[182]
woman (age 56)	ingestion of 5 wild castor seeds as treatment against constipation	recovered	Korea	detection of ricin in urine	[177]
Georgi Markov (age 49)	in 1978 assassination of Markov: poking with an umbrella for injection of a pellet with channels probably containing ricin	fatal	UK	suspected	[117, 204, 206, 217, 218]
Vladimir Kostov	in 1978 attempted assassination of Kostov: shot in his back with an air pistol for injection of a pellet with channels probably containing ricin	recovered	France	suspected	[117]
man (age 21)	ingestion of 30 castor seeds in attempting suicide, only some were masticated	recovered	France	detection of ricin in plasma and urine	[219]
woman (age 38)	in 1985 ingestion of 24 chopped castor seeds in attempting suicide	recovered	USA	circumstantial evidence	[165]
woman (age 20)	ingestion of 12 castor seeds in attempting suicide	recovered	Spain	circumstantial evidence	[220]
adolescent (age 16)	ingestion of 2 castor seeds in attempting suicide	recovered	USA	circumstantial evidence	[221]
man (age 20)	subcutaneous suicidal injection of castor seed extract	fatal	Poland	suspected	[183]
man (age 53)	chewing of 13 castor seeds. The mastication product was injected in attempting suicide	recovered	France	circumstantial evidence	[187]
man (age 61)	intention: suicide, injection of a solution of crushed castor seeds	fatal	USA	detection of ricinine in urine	[184, 222]
man (age 56), woman (age 59)	injection of extracted ricin from castor seeds into his wife and himself	fatal	Belgium	detection of ricin in urine and syringe	[186]
man (age 49)	i.v. and s.c. injection of castor seed extract in attempting suicide	fatal	Belgium	detection of ricinine in blood, urine, and syringe	[185]

^1^ Circumstantial evidence: the causative link to ricin intoxication is based on details of the case report, e.g., known or observed uptake of plant seeds, finding of plant material *etc.*; suspected: suspicion of ricin intoxication based on symptoms observed. ^2^ Table is organized by the publication date of literature cited. The table focuses on case reports including clinical signs, symptoms and treatment and makes no claim to be complete.

**Table 2:** Summary of animal intoxications with ricin.

Animal cases: dogs					
	Uptake/Ingestion	Outcome	Where	Detection and Diagnosis ^1^	Ref. ^2^
5 dogs	in 1977-1979 ingestion of organic fertilizer	3 fatal	Germany	circumstantial evidence	[223]
98 dogs	in 1989-2000 ingestion of unknown amount of castor seeds	7 fatal	USA	suspected	[224]
19 dogs	in 2001 ingestion of fertilizer containing castor seeds	7 fatal	Germany	circumstantial evidence; detection of ricin in fertilizer	[225]
dog	in 2002 ingestion of castor seed cakes used as fertilizer	fatal	Brazil	circumstantial evidence	[226]
dog	in 1999 ingestion of fertilizer based on castor seeds	recovered	Brazil	circumstantial evidence	[227]
dog	in 1999 ingestion of motor oil based on castor oil	recovered	Brazil	circumstantial evidence	[227]
35 dogs	in 2001-2003; details of intoxication not described	not described	USA	suspected	[188]
puppy	ingestion of unknown amount of castor beans	fatal	USA	detection of ricinine in stomach content	[228]
dog	ingestion of unknown amount of castor beans	recovered	Germany	circumstantial evidence	[229]
2 dogs	ingestion of fertilizer composed of *R. communis* material	fatal	Belgium	detection of ricinine in gastric and intestinal content , liver and kidney	[230]
15 dogs	in 2007 ingestion of soil conditioner with 10 % oil cake	13 fatal	Korea	suspected	[231]
9 dogs	in 2010, ingestion of fertilizer containing *R. communis*	2 fatal	Germany	detection of ricinine in urine and ricin in fertilizer and soil	this paper
different farm animals, mostly cows	in 1873 ingestion of flaxseed flour contaminated with castor seeds	recovered	Germany	circumstantial evidence	[232]
35 horses	in 1888 ingestion of flaxseed flour contaminated with castor seeds	1 fatal	Germany	circumstantial evidence	[233]
70 different animals	in 1950 ingestion of layers’ mash containing castor seed husks in meal	fatal 2 pigs, 1 heifer, 2 cattle	Ireland	circumstantial evidence	[234]
several 1000 ducks	in 1969-1971 ingestion of unknown amount of castor seeds	fatal for at least 10 ducks	USA	circumstantial evidence	[235]
1 horse	in 1999 ingestion and aspiration of ~2 L filtrate made of crushed castor seeds mixed with water	fatal	Brazil	suspected	[236]
45 sheep and goats	in 2005 ingestion of garden waste containing castor beans	fatal for 15 animals	Iran	circumstantial evidence	[237]

^1^ Circumstantial evidence: the causative link to ricin intoxication is based on details of the case report, e.g., known or observed uptake of plant seeds, finding of plant material *etc*.; suspected: suspicion of ricin intoxication based on symptoms observed. ^2^ Table is organized by the publication date of literature cited. The table focuses on case reports including clinical signs, symptoms and treatment and makes no claim to be complete.

## 7. Ricin Intoxications in Animals

Based on experimental poisoning of animals with *Ricinus communis* seeds, a variability in toxicity was observed. Whereas horses seem to be most sensitive, followed by geese, rodents and ruminants, chicken seem to be the most resistant animals [167,238]. Animals showed similar symptoms as humans after intoxication with ricin, that is weakness, profuse watery diarrhoea, dehydration with sunken eyes, dilation of pupils, depression, tachycardia, dyspnoea and colics. These signs and symptoms developed most frequently within 6-24 h. In biochemical examinations a high packed cell volume as a sign of severe dehydration and, as in humans, high activity of serum creatine kinase (CK) and AST as well as high concentrations of serum BUN and creatinine have been observed. Pathology of deceased animals also revealed gastroenteritis, necrosis and haemorrhage in heart and kidney [237]. In dogs, the most common clinical signs and symptoms included vomiting (80%), diarrhoea (37%), bloody diarrhoea (24%) and abdominal pain (14%). Biochemical parameters are similar to those in other animals [224].

While human cases of ricin poisoning mostly occur after ingestion of the unprocessed seeds, animal cases have also been described after uptake of processed castor seed products. After oil extraction, the press cake of the seeds is a rich source of protein, and is-after detoxification-used as cheap additive in organic fertilizer, soil conditioner or animal feed [11,13,21]. However, case reports from Europe, America and Asia describe poisoning of domestic animals, especially dogs, after ingestion of organic fertilizer containing castor cake (Table 2), leading to the hypothesis that the detoxification process itself is problematic and might leave residual active ricin within the press cake. In the majority of cases the amount of active ricin left after detoxification has not been quantified.

Considering the situation in Germany, dog poisoning in conjunction with organic fertilizer containing *R. communis* has been a problem over the last three decades. Since 1980, several independent cases have been described [223,225,229], and in our opinion there are a number of unreported cases which might not have been recognized. From 1980 until now, we found case reports on 34 poisoned dogs including 12 fatal cases in Germany (35%). In this context in 2001 all organic fertilizer-containing castor cake was temporarily taken off the German market, but was later re-introduced [225]. It is supposed that the fertilizer might be attractive for dogs due to admixing of castor cake with different organic additives [223].

**Figure 3:**
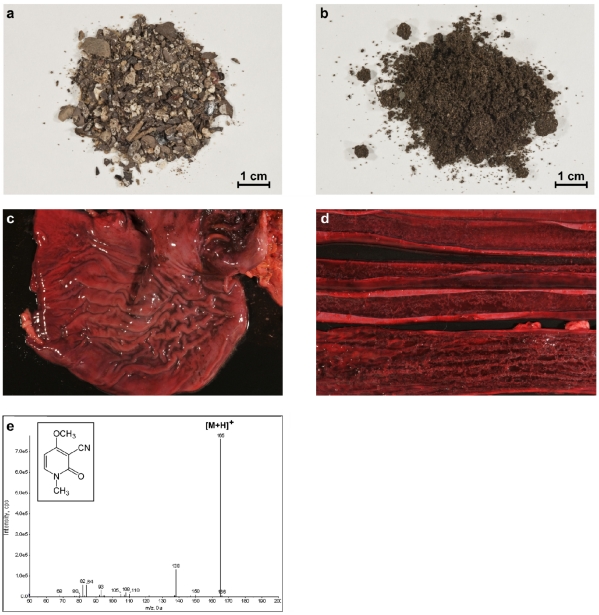
Postmortem analysis of a dog deceased after uptake of *R. communis*-containing fertilizer. (**a**) Sample of organic fertilizer which caused nine cases of ricin intoxication in dogs in Germany in March 2010; (**b**) Soil sample taken from a field which was treated with the fertilizer from (a). Both the fertilizer and the soil sample were shown to contain active ricin; (**c**) Stomach of the deceased dog showing marked oedema and hyperaemia; (**d**) Small intestine with acute fibrino-haemorrhagic enteritis; (**e**) LC-MS/MS spectrum of the dog’s urine containing ricinine. The chemical formula of ricinine is given in the inset (molecular weight 164.2 g/mol); the peak at *m*/*z* = 165 represents the protonated precursor ion ([M+H]^+^).

Exemplarily we briefly describe a recent case that occurred in Dormagen, Germany, in 2010: 9 dogs fell ill after ingestion of unknown amounts of organic fertilizer freshly distributed on a local field (Figure 3a,b). The dogs were suffering from vomiting, abdominal pain and haemorrhagic diarrhoea, and one dog died and another dog was euthanized about 48 h after ingestion. One animal was submitted for necropsy. Macroscopically, the stomach showed marked oedema (Figure 3c), and within the dog’s small intestine an acute fibrino-haemorrhagic enteritis was identified (Figure 3d). Laboratory analysis revealed 1715 µg/g ricin in the fertilizer and 380 and 820 µg/g ricin in two soil samples taken from the manured field using a ricin-specific ELISA detecting the active toxin (Pauly *et al.*, manuscript in preparation). For comparison, in the case described by Ebbecke *et al.* in 2001 up to 10 µg/g active ricin was detected in fertilizer samples [225].

Based on these results, one of the dogs was thoroughly analyzed for traces of ricin and ricinine in different organs and urine. While it was technically not possible to detect ricin or ricin DNA in samples taken from kidney, liver, stomach and blood, ricinine was unambiguously detected in the urine of the deceased animal using LC-MS/MS techniques and multiple reaction monitoring (Figure 3e). To our knowledge, this was the first German case showing a causative link between the ingestion of ricin-contaminated fertilizer and a fatal outcome of the poisoning, with ricinine being detected in the dog’s urine as a surrogate marker for uptake of *R. communis*-material. Fatal cases in dogs after ingestion of fertilizer or soil conditioner have been reported before from USA, Brazil, Korea and Belgium (Table 2; [188,226,230,231]). In most of the cases, the link to *R. communis* was suspected based on the clinical symptoms or on circumstantial evidence, e.g., observed uptake of plant seeds (Table 2). However, in the case of dog poisoning in Belgium, ricinine as surrogate marker was successfully detected in liver, kidney and gastric and intestinal content [230]. Similarly, Mouser *et al.* detected ricinine in the stomach content of a dog which had ingested an unknown amount of castor beans [228].

Animal intoxications did also occur in the past due to incorrectly processed feed containing *R. communis* material. In former times, intoxications of farm animals (horses, ruminants) were reported after uptake of flaxseed flour contaminated with castor seeds [232,233]. More recently, in Iran, sheep and goats were poisoned after ingestion of garden waste containing castor seeds [237]. However, nowadays accidental poisoning of animals due to castor plant-contaminated feed is rare, most likely because of different national and international regulations which limit the amount of *R. communis* in animal feed. As an example, within the European Union the Commission Directive 2009/141/EC states *R. communis* as an undesirable substance in animal feed, with a maximum content of 10 mg/kg seeds and husks from the plant allowed relative to animal feed with a moisture content of 12% [239].

In contrast to the existing regulations on animal feed, to our knowledge there is no international regulation limiting the amount of *R. communis* in fertilizer. However, there are national regulations, e.g., in Germany the so-called fertilizer regulation which allows *R. communis*-residual material in fertilizer if no acute oral toxicity in rats is observed after uptake of 2000 mg material per kg body weight (*Düngemittelverordnung* -DüMV-, Attachment 2, Nr. 7.1.5; [240]). Currently this regulation is under evaluation, based on the recent cases of dog poisoning described. In this context we independently tested several samples of organic fertilizer from different brands and found significant concentrations of active ricin (up to 3000 µg/g fertilizer), corroborating the hypothesis that the detoxification process of castor cake is not always thoroughly performed and controlled. Therefore, it is planned to update the German fertilizer regulation to state a definite amount of ricin maximally allowed in fertilizer. Since the problem is not restricted to Germany or Europe, international regulations should be established to agree on a limit of ricin maximally allowed in fertilizer. To our understanding, this limit should not be based on animal oral toxicities (because of ethical concerns in animal testing and the variable toxicity in animals), but on the detectable amount of active ricin per kg fertilizer.

In summary, veterinary cases of ricin poisoning occurred in different animal species, mostly in domestic animals. Intoxications of animals were caused either by the unprocessed plant seeds or by processed castor cake as it is used as by-product in organic fertilizer, calling for international regulations which clearly limit the amount of ricin in fertilizer. In contrast to humans, poisoning of animals is statistically less well surveyed. Nevertheless, fatality rates have been estimated: from 98 cases of dog poisoning, Albretsen *et al.* deduced a fatality rate of about 7% [224]. Based on all cases of dog poisoning listed in Table 2 we found a higher fatality rate of 35.3% for Germany and 23.5% world-wide (Figure 2B). In humans, however, Rauber *et al*. reported a fatality rate of 1.8% for 751 cases observed [165]. Based on all human accidental intoxications listed in Table 1, we found a similar fatality rate of 1.5% (Figure 2A). However, among the limited number of intended human poisonings reported (Table 1), the observed fatality rate was much higher (45.5%). Therefore, the parenteral uptake of ricin leads to a more severe outcome than the oral uptake, as has been expected from animal experiments. Furthermore, when comparing fatality rates in human and veterinary cases, one might be tempted to speculate that ricin has an increased toxicity in dogs compared to humans (Figure 2). However, sound toxicity data for humans and dogs do not exist and additional factors might play a role, like adequate and timely treatment of animals or higher accessibility of the toxin from the crushed fertilizer material.

## 8. Detection of Ricin or *Ricinus communis*

In “naturally” occurring cases, the primary diagnosis is based on the case history reported and on clinical symptoms. Since ricin induces unspecific symptoms also observed with many other diseases, the diagnosis might be difficult as long as the suspicious matter is not identified, e.g., seeds found in vomit, intestine or faeces. In any case, laboratory detection is a necessary tool to confirm intoxication with *R. communis* in clinical samples and to screen for the source of intoxication in environmental samples (e.g., fertilizer, soil) or food samples.

Among the different detection methods available, antibody-based immunoassays belong to the standard technologies applied to detect and to quantify ricin in clinical and environmental samples as well as in food and feed. Enzyme-linked immunosorbent assays (ELISA) have been developed by different groups [132,135,241,242,243,244,245,246,247,248,249,250,251,252]. Some of them are able to quantify ricin with detection limits down to a few pg/mL (limit of detection: 2 pg/mL [246] and 40 pg/mL [253]). ELISA-based methods have been successfully used to track ricin in tissues after experimental intoxication [132,135,142,244,245,251]. Traditional chromogenic substrates have been replaced by electro chemi luminescence [248,253], electrochemical [249] or PCR read-out [132,254,255,256] in order to increase sensitivity and to reduce background signal noise, with the most sensitive detection limit of 10 fg/mL given for an immuno-PCR approach [254]. Immuno-PCR detection of ricin was used to measure ricin out of food matrices and to follow the fate of ricin after experimental intoxication [132,254]. Most ELISA require several hours to perform, meaning that valuable time is lost before countermeasures can be implemented-this is especially important in case of intentional or criminal use of ricin in a potential bioterrorism scenario. This issue was addressed by the development of faster (<1 h) assays based on fiber-optic sensors or rapid electrical detection [249,257]. Furthermore, immunochromatographic and lateral-flow assays (LFA) have been developed to meet the demand for fast and technically easy on-site detection [248,258]. LFA are usually around 1,000 times less sensitive than standard ELISA and reach detection limits of 1-50 ng/mL [119,253,259,260].

Apart from antibodies, DNA- or RNA-aptamers have been reported to selectively bind ricin [261,262,263,264,265,266,267,268]. It has been proposed that they could be used as an alternative to antibody-based detection methods [269], but still their diagnostic value in protein detection, especially out of complex matrices, is limited. To our knowledge, only one assay based on aptamer technology for detection of ricin (B-chain) out of beverages has been published, and a detection limit of 25 ng/mL for intact ricin was reported [268].

One drawback of all antibody- and aptamer-based assays is that they do not unambiguously detect their target molecule, meaning that cross-reactivity to related antigens or high concentrations of interfering substances might lead to false positive results. Furthermore, the discrimination of different ricin isoforms and/or *R. communis* agglutinin is technically not feasible. For unambiguous detection of ricin and its selective discrimination from *R. communis* agglutinin, sequence information is necessary. Modern state-of-the-art mass spectrometry technologies are able to deliver information on the target’s protein sequence and its glycosylation pattern: highly sophisticated technologies like electrospray ionisation (ESI) or matrix-assisted laser-desorption/ionisation time-of-flight (MALDI-TOF) mass spectrometry (MS) as well as liquid chromatography (LC)-MS/MS analysis of the tryptic peptide fragments have been developed to unequivocally identify ricin out of crude toxin preparations [270,271,272,273,274] and to analyze its glycosylation pattern [118,124]. However, limited sensitivity and the difficulty to identify ricin out of complex matrices lead to the combination of immunoaffinity enrichment with MS-based detection. This combination has been successfully applied to the detection of ricin out of different complex matrices [115,275,276,277,278,279], yielding a detection limit of down to 0.64 ng/mL [278].

While the above-mentioned technologies are very useful to detect the presence of ricin, they lack the ability to measure the functional activity of the toxin, *i.e.*, the ability to discriminate inactive (non-hazardous) versus active (hazardous) material. This point is important in the case of an intentional release of ricin, especially with regard to emergency operating schedules, forensic analysis and therapy. The discrepancy between presence of the ricin protein and lack of toxicity has been noted for some immunoassays [280], while in other assays detection seems to correlate with activity [281,282,283]. Functional assays for ricin have traditionally been done by animal toxicity tests and *in vitro* cytotoxicity assays [130,284]. Later, cytotoxicity assays have been amended to detect ricin out of complex matrices [282,283,285,286]. Using functionally blocking antibodies, these tests enable the discrimination of ricin from other cytotoxins. The ability of ricin to inactivate ribosome activity was elucidated in the 1970s [287,288], leading to the first functional cell-free *in vitro* assay [55,289] which is in principle still in use for ricin and other RIPs [290,291]. After the molecular mechanism of depurination was deciphered [35,36], a number of methods assaying the functional activity of the A-chain were developed. The single adenine released by the A-chain was detected by different methods including HPLC, MS, fluorescence, RT-PCR or enzymatic reaction [121,292,293,294,295,296,297]. Since adenine might be present in biological samples or be released by unspecific enzymes or other RIPs, it was found to be superior first to separate ricin from the matrix by an immunocapture step, followed by mass-spectrometric detection of either the released adenine or the depurinated substrate [115,277,278,298]. These sophisticated MS-based functional assays have been shown to detect ricin from environmental or clinical matrices. These methods combine the measurement of functional activity with the discriminatory power of MS for the identification of ricin, resulting in a very powerful technology for the detection and functional characterization of ricin out of complex matrices.

While different ricin detection methods were successfully applied to detect ricin in complex matrices (also environmental and food matrices involved in real cases), the detection of the toxin itself in clinical samples has been difficult in real cases. As shown in Table 1 and Table 2, only in three reports has ricin been detected in urine or plasma of patients [177,186,219]. The problem with ricin detection in forensic analysis is that the molecule is obviously rapidly absorbed within the tissue and internalized into the cells, limiting the time window of detection as shown by research in animals. Deduced from experimental intoxication of animals, orally applied ricin passes through stomach and small intestine within 24 h. Most of the ricin has reached the large intestine by 12 h where it can be detected by immunoassays for up to 72 h [299]. From 24 h onwards substantial amounts are found within feces. Up to 50% of the applied ricin seems to be absorbed or no longer be available for detection [299]. In a similar study, orally administered ricin could soon be detected in faeces (2-24 h), but some ricin reaches the blood from which it is quickly absorbed by different tissues [132]. The liver and spleen seem to be the most prominent targets, but the total amount of detectable ricin is very small compared to the amount applied [142]. In light of the available data it seems reasonable to suggest that the majority of orally ingested ricin is destroyed in the stomach and a fair amount is shed with the faeces. Only a small proportion seems to reach the bloodstream and the inner organs. In the liver, phagocytotic Kupffer cells and sinusoidal endothelial cells have been reported to be the main targets [300,301,302,303,304]. Indeed, hepatic Kupffer and sinusoidal endothelial cells as well as other phagocytotic cells (e.g., macrophages, granulocytes, dendritic cells) constitute the forefront in immunological defense and do not only express glycolipids and glycoproteins on their cell surface, but are also equipped with lectin receptors which enable the rapid uptake of ricin into the cells [305,306].

As the detection of ricin in real cases is difficult, ricinine has successfully been used as surrogate marker in six human and veterinary cases reported so far (Tables 1 and 2). The advantage of ricinine biomonitoring stems from the small size of the molecule which can be easily extracted and monitored by chromatographic and MS-based methods. Animal studies have shown that ricinine can be detected in urine for up to 48 h after exposure in rats [222].

Initially, ricinine was detected using paper chromatography, UV detection [307,308] and later liquid chromatography (LC) [309] or combinations of LC or gas chromatography (GC) with MS. The latter gave superior results and allowed to identify ricinine in crude ricin preparations [310]. Solvent- or solid-phase extraction were applied to extract ricinine from food, feed or clinical samples [185,228,311]. By using an isotope-labeled ricinine as an internal standard, quantification of the molecule became possible [222,312]. The molecule is co-extracted with ricin from the seeds and can be easily detected in crude extracts of *R. communis*. Therefore, oral intoxications with *Ricinus communis* seeds have been successfully confirmed by the detection of ricinine from urine, blood, liver, kidney or gastric content in human and veterinary cases [182,185,222,228,230].

## 9. Treatment and Vaccination

Currently, no approved specific therapy or antidote against ricin intoxication is available. The treatment focuses on supportive medicine and involves application of intravenous fluids and suppression of hypertension. To prevent further absorption of the toxin, treatment with activated charcoal or gastric lavage have been used depending on the time of admission after oral ingestion [111].

Several tracks have been followed to identify therapeutic molecules against ricin intoxication, like antibodies, small molecule inhibitors, aptamers and sugars [313,314]. So far, antibodies are the only class of molecules showing real promise [128,315,316]. Basically, the concept goes back to the seminal work of Paul Ehrlich who showed in his work on anti-toxins that animals can be immunized against ricin intoxication and that blood from these animals can transfer protection to other animals [317]. The fruitful cooperation with Emil von Behring, Shibasaburo Kitasato and Robert Koch laid the foundation for the serum therapy and vaccination. Throughout recent years, ricin-specific antisera or polyclonal antibodies (pAb) have been generated in different species (e.g., rabbit, goat, sheep, chicken and even humans) and tested as post-exposure therapeutic in animal models [128,137,318,319,320,321,322,323,324]. For ricin it seems that protection can be conferred by antibodies when given concurrently or within 10 h after intoxication, depending on the route of toxin uptake [128,132]. Once ricin has been internalized into the cells, it cannot be inactivated by antibodies, limiting the therapeutic window. In order to circumvent the side effects of animal antisera (anaphylaxis, serum sickness), recent research focused on humanized monoclonal antibodies or recombinant antibodies [325,326].

Apart from antibodies, different small molecules, glycostructures and aptamers have been approached and tested *in vitro*, but so far lacking convincing animal studies to show protection *in vivo* [267,313,327,328,329,330]. However, recently, small molecule inhibitors of intracellular retrograde ricin transport have been identified, one of which imparted protection to mice [331].

In order to protect selected persons at risk, e.g., military personnel and emergency service staff, there has been an interest in developing a vaccine against ricin intoxication. While there is no such vaccine readily available, one is in an advanced stage of legislative approval [332,333,334,335]: RiVax^TM^ is based on a recombinant catalytic inactive A-chain of ricin and was subjected to pilot clinical trials in humans where it was shown to induce functionally active antibodies.

## 10. Conclusion

*Ricinus communis* is of economic interest for the production of castor oil and the numerous industrial, medical and cosmetic products derived thereof. After detoxification, the defatted press cake of the oil production is industrially used as a by-product of organic fertilizer and as low-value feed. For a long time, intoxications of humans and animals with *R. communis* have been known, caused by the main toxic component of the plant, ricin. Among all plant poisonings reported, human cases of ricin poisoning are rare and the fatality rate is-based on modern supportive care-low (around 1.8% [165]), except for suicide cases where a ricin-containing extract is injected, reflecting the higher toxicity of ricin after parenteral application compared to oral uptake. Although cases of animal poisoning are less well surveyed, intoxications of animals, especially dogs, have been observed either by the unprocessed plant seeds or by processed castor cake products. Recent cases from Europe, Asia and America linked to *R. communis*-containing fertilizer show that the detoxification of castor cake is obviously problematic and not always thoroughly performed, calling for international regulations and stringent control to clearly limit the amount of ricin in fertilizer. This is even more important as the fatality rate in dogs-based on the limited number of cases available-seems to be higher than in humans (between 7 and 35%; Table 2 and [224]). With respect to forensic analysis, ricin detection in clinical samples is difficult due to its rapid absorption and internalization within the tissue. As a surrogate marker, the alkaloid ricinine can be successfully monitored.

## References

[1] Scarpa A. Guerci A. Various uses of the castor oil plant (*Ricinus communis* L.). A review. J. Ethnopharmacol. 1982g 5 117 137 7035750 10.1016/0378-8741(82)90038-1

[2] Serpico M., White R., Nicholson P.T., Shaw I. (2000). Oil, Fat and Wax.. Ancient Egyptian Materials and Technology.

[3] Weiss E.A. (2000). Oilseed Crops.

[4] Ogunniyi D.S. (2006). Castor oil: A vital industrial raw material.. Bioresour. Technol..

[5] Mutlu H., Meier M.A.R. (2010). Castor oil as a renewable resource for the chemical industry.. Eur. J. Lipid Sci. Technol..

[6] Parkouda C., Nielsen D.S., Azokpota P., Ouoba L.I.I., Amoa-Awua W.K., Thorsen L., Hounhouigan J.D., Jensen J.S., Tano-Debrah K., Diawara B. (2009). The microbiology of alkaline-fermentation of indigenous seeds used as food condiments in Africa and Asia.. Crit. Rev. Microbiol..

[7] Odunfa S.A. (1985). Microbiological and toxicological aspects of fermentation of castor oil seeds for ogiri production.. J. Food Sci..

[8] De Lima da Silva N., Maciel M., Batistella C., Filho R. (2006). Optimization of biodiesel production from castor oil.. Appl. Biochem. Biotechnol..

[9] Berman P., Nizri S., Wiesman Z. (2011). Castor oil biodiesel and its blends as alternative fuel.. Biomass Bioenerg..

[10] Behl C.R., Pande M.B., Pande D.P., Radadia M.S.  (1986). Nutritive value of matured wilted castor (*Ricinus communis* Linn.) leaves for crossbred sheep.. Indian J. Anim. Sci..

[11] Robb J.G., Laben R.C., Walker H.G., Herring V. (1974). Castor meal in dairy rations. J. Dairy Sci..

[12] Gowda N.K.S., Pal D.T., Bellur S.R., Bharadwaj U., Sridhar M., Satyanarayana M.L., Prasad C.S., Ramachandra K.S., Sampath K.T. (2009). Evaluation of castor (*Ricinus communis*) seed cake in the total mixed ration for sheep.. J. Sci. Food Agric..

[13] Balogun J.K., Auta J., Abdullahi S.A., Agboola O.E. Potentials of Castor Seed Meal (*Ricinus communis* L.) as Feed Ingredient for Oreochromis Niloticus.. Proceedings of the 19th Annual Conference Fisheries Society Nigeria.

[14] Diniz L.L., Valadares Filho S.C., de Oliveira A.S., Pina D.S., de Lima da Silva N., Benedeti P.B., Baião G.F., Campos J.M.S., Valadares R.F.D. (2011). Castor bean meal for cattle finishing: 1-nutritional parameters.. Livest. Sci..

[15] Vilhjalmsdottir L., Fisher H. (1971). Castor bean meal as a protein source for chickens: Detoxification and determination of limiting amino acids.. J. Nutr..

[16] Ani A.O. (2007). Effects of graded levels of dehulled and cooked castor oil bean (*Ricinus communis* L.) meal and supplementary L-lysine on performance of broiler finishers.. J. Trop. Agric. Food Environ. Ext..

[17] Tangl H. (1939). On the feeding value of extracted castor-oil meal.. Kiserletuegyi Koezlemenyek.

[18] Alexander J., Benford D., Cockburn A., Cravedi J.P., Dogliotti E., di Domenico A., Férnandez-Cruz M.L., Fürst P., Fink-Gremmels J., Galli C.L. (2008). Scientific opinion of the panel on contaminants in the food chain on a request from the European commision on ricin (from *Ricinus communis*) as undesirable substances in animal feed.. EFSA J..

[19] Barnes D.J., Baldwin B.S., Braasch D.A. (2009). Degradation of ricin in castor seed meal by temperature and chemical treatment.. Ind. Crops Prod..

[20] Gupta A.P., Antil R.S., Narwal R.P. (2004). Utilization of deoiled castor cake for crop production.. Arch. Agron. Soil Sci..

[21] Lima R.L.S., Severino L.S., Sampaio L.R., Sofiatti V., Gomes J.A., Beltrão N.E.M. (2011). Blends of castor meal and castor husks for optimized use as organic fertilizer.. Ind. Crops Prod..

[22] (2007). Final report on the safety assessment of *Ricinus communis* (castor) seed oil, hydrogenated castor oil, glyceryl ricinoleate, glyceryl ricinoleate se, ricinoleic acid, potassium ricinoleate, sodium ricinoleate, zinc ricinoleate, cetyl ricinoleate, ethyl ricinoleate, glycol ricinoleate, isopropyl ricinoleate, methyl ricinoleate, and octyldodecyl ricinoleate. Int. J. Toxicol..

[23] Anandan S., Kumar G.K.A., Ghosh J., Ramachandra K.S. (2005). Effect of different physical and chemical treatments on detoxification of ricin in castor cake.. Anim. Feed Sci. Technol..

[24] Gandhi V., Cherian K., Mulky M. (1994). Detoxification of castor seed meal by interaction with sal seed meal.. J. Am. Oil Chem. Soc..

[25] Puttaraj S., Bhagya S., Murthy K.N., Singh N. (1994). Effect of detoxification of castor seed (*Ricinus communis*) protein isolate on its nutritional quality.. Plant Food Hum. Nutr..

[26] Melo W.C., dos Santos A.S., Santa Anna L.M.M., Pereira N. (2008). Acid and enzymatic hydrolysis of the residue from castor bean (*Ricinus communis* L.) oil extraction for ethanol production: Detoxification and biodiesel process integration.. J. Braz. Chem. Soc..

[27] De Oliveira A.S., Campos J.M.S., Oliveira M.R.C., Brito A.F., Filho S.C.V., Detmann E., Valadares R.F.D., de Souza S.M., Machado O.L.T. (2010). Nutrient digestibility, nitrogen metabolism and hepatic function of sheep fed diets containing solvent or expeller castor seed meal treated with calcium hydroxide. Anim. Feed Sci. Technol..

[28] Auld D.L., Rolfe R.D., McKeon T.A. (2001). Development of castor with reduced toxicity.. J. New Seeds.

[29] Knapp O. (1943). Versuche zur Züchtung einer giftfreien Ricinussorte.. Theor. Appl. Genet..

[30] Lowery C.C., Auld D.L., Rolfe R., McKeon T.A., Goodrum J., Janick J., Whipkey A. (2002). Barriers to Commercialization of a Castor Cultivar with Reduced Concentration of Ricin. Issues in New Crops and New Uses.

[31] Auld D.L., Pinkerton S.D., Boroda E., Lombard K.A., Murphy C.K., Kenworthy K.E., Becker W.D., Rolfe R.D., Ghetie V. (2003). Registration of TTU-LRC castor germplasm with reduced levels of ricin and RCA120.. Crop Sci..

[32] Kobert R. (1906). Lehrbuch der Intoxikationen.

[33] Stillmark H. (1888). Ueber Ricin, ein giftiges Fragment aus den Samen von Ricinus comm. L und einigen anderen Euphorbiaceen.

[34] Bradberry S.M., Dickers K.J., Rice P., Griffiths G.D., Vale J.A. (2003). Ricin poisoning.. Toxicol. Rev..

[35] Endo Y., Mitsui K., Motizuki M., Tsurugi K. (1987). The mechanism of action of ricin and related toxic lectins on eukaryotic ribosomes. The site and the characteristics of the modification in 28S ribosomal RNA caused by the toxins.. J. Biol. Chem..

[36] Endo Y., Tsurugi K. (1987). RNA *N-*glycosidase activity of ricin A-chain. Mechanism of action of the toxic lectin ricin on eukaryotic ribosomes.. J. Biol. Chem..

[37] Lord J.M., Spooner R.A. (2011). Ricin trafficking in plant and mammalian cells.. Toxins.

[38] Lappi D.A., Kapmeyer W., Beglau J.M., Kaplan N.O. (1978). The disulfide bond connecting the chains of ricin.. Proc. Natl. Acad. Sci. USA.

[39] Baenziger J.U., Fiete D. (1979). Structural determinants of *Ricinus communis* agglutinin and toxin specificity for oligosaccharides.. J. Biol. Chem..

[40] Sandvig K., van Deurs B. (1999). Endocytosis and intracellular transport of ricin: Recent discoveries.. FEBS Lett..

[41] Olsnes S., Saltvedt E., Pihl A. (1974). Isolation and comparison of galactose-binding lectins from *Abrus precatorius* and *Ricinus communis*.. J. Biol. Chem..

[42] Olsnes S., Pihl A. (1973). Different biological properties of the two constituent peptide chains of ricin, a toxic protein inhibiting protein synthesis. Biochemistry.

[43] Lin T.T.S., Li S.S.L. (1980). Purification and physicochemical properties of ricins and agglutinins from *Ricinus communis*.. Eur. J. Biochem..

[44] Nicolson G.L., Blaustein J. (1972). The interaction of *Ricinus communis* agglutinin with normal and tumor cell surfaces.. Biochim. Biophys. Acta Biomembr..

[45] Tomita M., Kurokawa T., Onozaki K., Ichiki N., Osawa T., Ukita T. (1972). Purification of galactose-binding phytoagglutinins and phytotoxin by affinity column chromatography using sepharose.. Cell Mol. Life Sci..

[46] Roberts L.M., Lamb F.I., Pappin D.J., Lord J.M. (1985). The primary sequence of *Ricinus communis* agglutinin. Comparison with ricin.. J. Biol. Chem..

[47] Araki T., Funatsu G. (1987). The complete amino acid sequence of the B-chain of ricin E isolated from small-grain castor bean seeds. Ricin E is a gene recombination product of ricin D and *Ricinus communis* agglutinin.. Biochim. Biophys. Acta.

[48] Ladin B.F., Murray E.E., Halling A.C., Halling K.C., Tilakaratne N., Long G.L., Houston L.L., Weaver R.F. (1987). Characterization of a cDNA encoding ricin E, a hybrid ricin-*Ricinus communis* agglutinin gene from the castor plant *Ricinus communis*. Plant Mol. Biol..

[49] Mise T., Funatsu G., Ishiguro M., Funatsu M. (1977). Isolation and characterization of ricin E from castor beans.. Agric. Biol. Chem..

[50] Lord J., Roberts L., Robertus J. (1994). Ricin: Structure, mode of action, and some current application. FASEB J..

[51] Sweeney E.C., Tonevitsky A.G., Temiakov D.E., Agapov I.I., Saward S., Palmer R.A. (1997). Preliminary crystallographic characterization of ricin agglutinin.. Proteins Struct. Funct. Bioinf..

[52] Brandt N.N., Chikishev A.Y., Sotnikov A.I., Savochkina Y.A., Agapov I.I., Tonevitskii A.G., Kirpichnikov M.P. (2001). Conformational difference between ricin and ricin agglutinin in solution and crystal.. Dokl. Biochem. Biophys..

[53] Saltvedt E. (1976). Structure and toxicity of pure ricinus agglutinin.. Biochim. Biophys. Acta.

[54] Cawley D.B., Hedblom M.L., Houston L.L. (1978). Homology between ricin and *Ricinus communis* agglutinin: Amino terminal sequence analysis and protein synthesis inhibition studies.. Arch. Biochem. Biophys..

[55] Olsnes S., Fernandez-Puentes C., Carrasco L., Vazquez D. (1975). Ribosome inactivation by the toxic lectins abrin and ricin.. Eur. J. Biochem..

[56] Zhan J., Zhou P. (2003). A simplified method to evaluate the acute toxicity of ricin and ricinus agglutinin.. Toxicology.

[57] Lord J.M., Harley S.M. (1985). *Ricinus communis* agglutinin B chain contains a fucosylated oligosaccharide side chain not present on ricin B chain.. FEBS Lett..

[58] Sandvig K., Torgersen M.L., Engedal N., Skotland T., Iversen T.-G. (2010). Protein toxins from plants and bacteria: Probes for intracellular transport and tools in medicine.. FEBS Lett..

[59] Sandvig K., van Deurs B. (2002). Membrane traffic exploited by protein toxins.. Annu. Rev. Cell Dev. Biol..

[60] Lord J.M., Roberts L.M., Lencer W.I. (2006). Entry of protein toxins into mammalian cells by crossing the endoplasmic reticulum membrane: Co-opting basic mechanisms of endoplasmic reticulum-associated degradation.. Curr. Top. Microbiol. Immunol..

[61] Spooner R.A., Smith D.C., Easton A.J., Roberts L.M., Lord J.M. (2006). Retrograde transport pathways utilised by viruses and protein toxins.. Virol. J..

[62] Moya M., Dautry-Varsat A., Goud B., Louvard D., Boquet P. (1985). Inhibition of coated pit formation in HEp2 cells blocks the cytotoxicity of diphtheria toxin but not that of ricin toxin.. J. Cell Biol..

[63] Shurety W., Bright N.A., Luzio J.P. (1996). The effects of cytochalasin D and phorbol myristate acetate on the apical endocytosis of ricin in polarised Caco-2 cells.. J. Cell Sci..

[64] Iversen T.-G., Skretting G., Llorente A., Nicoziani P., van Deurs B., Sandvig K. (2001). Endosome to Golgi transport of ricin is independent of clathrin and of the Rab9- and Rab11-GTPases.. Mol. Biol. Cell.

[65] Llorente A., Rapak A., Schmid S.L., van Deurs B., Sandvig K. (1998). Expression of mutant dynamin inhibits toxicity and transport of endocytosed ricin to the Golgi apparatus.. J. Cell Biol..

[66] Jackman M.R., Shurety W., Ellis J.A., Luzio J.P. (1994). Inhibition of apical but not basolateral endocytosis of ricin and folate in Caco-2 cells by cytochalasin D.. J. Cell Sci..

[67] Jackman M.R., Ellis J.A., Gray S.R., Shurety W., Luzio J.P. (1999). Cell polarization is required for ricin sensitivity in a Caco-2 cell line selected for ricin resistance.. Biochem. J..

[68] Van Deurs B., Sandvig K., Petersen O., Olsnes S., Simons K., Griffiths G. (1988). Estimation of the amount of internalized ricin that reaches the trans-Golgi network.. J. Cell Biol..

[69] van Deurs B., Tønnessen T.I., Petersen O.W., Sandvig K., Olsnes S. (1986). Routing of internalized ricin and ricin conjugates to the Golgi complex.. J. Cell Biol..

[70] Grimmer S., Iversen T.-G., van Deurs B., Sandvig K. (2000). Endosome to Golgi transport of ricin is regulated by cholesterol.. Mol. Biol. Cell.

[71] Lauvrak S.U., Llorente A., Iversen T.-G., Sandvig K. (2002). Selective regulation of the Rab9-independent transport of ricin to the Golgi apparatus by calcium.. J. Cell Sci..

[72] Tjelle T.E., Brech A., Juvet L.K., Griffiths G., Berg T. (1996). Isolation and characterization of early endosomes, late endosomes and terminal lysosomes: Their role in protein degradation. J. Cell Sci..

[73] Dang H., Klokk T.I., Schaheen B., McLaughlin B.M., Thomas A.J., Durns T.A., Bitler B.G., Sandvig K., Fares H. (2011). Derlin-dependent retrograde transport from endosomes to the Golgi apparatus.. Traffic.

[74] Llorente A., Lauvrak S.U., van Deurs B., Sandvig K. (2003). Induction of direct endosome to endoplasmic reticulum transport in chinese hamster ovary (CHO) cells (LDLF) with a temperature-sensitive defect in ε-coatomer protein (ε-Cop).. J. Biol. Chem..

[75] Amessou M., Fradagrada A., Falguières T., Lord J.M., Smith D.C., Roberts L.M., Lamaze C., Johannes L. (2007). Syntaxin 16 and syntaxin 5 are required for efficient retrograde transport of several exogenous and endogenous cargo proteins.. J. Cell Sci..

[76] Rapak A., Falnes P.O., Olsnes S. (1997). Retrograde transport of mutant ricin to the endoplasmic reticulum with subsequent translocation to cytosol.. Proc. Natl. Acad. Sci. USA.

[77] Majoul I., Sohn K., Wieland F.T., Pepperkok R., Pizza M., Hillemann J., Söling H.-D. (1998). KDEL receptor (Erd2p)-mediated retrograde transport of the cholera toxin A subunit from the Golgi involves Copi, p23, and the COOH terminus of Erd2. J. Cell Biol..

[78] Lee M.C.S., Miller E.A., Goldberg J., Orci L., Schekman R. (2004). Bi-directional protein transport between the ER and Golgi.. Annu. Rev. Cell Dev. Biol..

[79] Spooner R.A., Watson P.D., Marsden C.J., Smith D.C., Moore K.A.H., Cook J.P., Lord J.M., Roberts L.M. (2004). Protein disulphide-isomerase reduces ricin to its A and B chains in the endoplasmic reticulum.. Biochem. J..

[80] Bellisola G., Fracasso G., Ippoliti R., Menestrina G., Rosén A., Soldà S., Udali S., Tomazzolli R., Tridente G., Colombatti M. (2004). Reductive activation of ricin and ricin A-chain immunotoxins by protein disulfide isomerase and thioredoxin reductase.. Biochem. Pharmacol..

[81] Deeks E.D., Cook J.P., Day P.J., Smith D.C., Roberts L.M., Lord J.M. (2002). The low lysine content of ricin A chain reduces the risk of proteolytic degradation after translocation from the endoplasmic reticulum to the cytosol.. Biochemistry.

[82] Di Cola A., Frigerio L., Lord J.M., Ceriotti A., Roberts L.M. (2001). Ricin A chain without its partner B chain is degraded after retrotranslocation from the endoplasmic reticulum to the cytosol in plant cells.. Proc. Natl. Acad. Sci. USA.

[83] Li S., Spooner R.A., Allen S.C.H., Guise C.P., Ladds G., Schnoder T., Schmitt M.J., Lord J.M., Roberts L.M. (2010). Folding-competent and folding-defective forms of ricin A chain have different fates after retrotranslocation from the endoplasmic reticulum.. Mol. Biol. Cell.

[84] Meusser B., Hirsch C., Jarosch E., Sommer T. (2005). ERAD: The long road to destruction.. Nat. Cell Biol..

[85] Simpson J.C., Roberts L.M., Romisch K., Davey J., Wolf D.H., Lord J.M. (1999). Ricin A chain utilises the endoplasmic reticulum-associated protein degradation pathway to enter the cytosol of yeast.. FEBS Lett..

[86] Słomińska-Wojewódzka M., Gregers T.F., Wälchli S., Sandvig K. (2006). EDEM is involved in retrotranslocation of ricin from the endoplasmic reticulum to the cytosol.. Mol. Biol. Cell.

[87] Sokołowska I., Wälchli S., Węgrzyn G., Sandvig K., Słomińska-Wojewódzka M. (2011). A single point mutation in ricin A-chain increases toxin degradation and inhibits EDEM1-dependent ER retrotranslocation.. Biochem. J..

[88] Spooner R.A., Hart P.J., Cook J.P., Pietroni P., Rogon C., Höhfeld J., Roberts L.M., Lord J.M. (2008). Cytosolic chaperones influence the fate of a toxin dislocated from the endoplasmic reticulum.. Proc. Natl. Acad. Sci. USA.

[89] Chiou J.-C., Li X.-P., Remacha M., Ballesta J.P.G., Tumer N.E. (2008). The ribosomal stalk is required for ribosome binding, depurination of the rRNA and cytotoxicity of ricin A chain in *Saccharomyces cerevisiae*. Mol. Microbiol..

[90] Lord M.J., Jolliffe N.A., Marsden C.J., Pateman C.S.C., Smith D.C., Spooner R.A., Watson P.D., Roberts L.M. (2003). Ricin: Mechanisms of cytotoxicity.. Toxicol. Rev..

[91] Dai J., Zhao L., Yang H., Guo H., Fan K., Wang H., Qian W., Zhang D., Li B., Wang H. (2011). Identification of a novel functional domain of ricin responsible for its potent toxicity.. J. Biol. Chem..

[92] Jetzt A.E., Cheng J.-S., Tumer N.E., Cohick W.S. (2009). Ricin A-chain requires c-Jun *N-*terminal kinase to induce apoptosis in nontransformed epithelial cells.. Int. J. Biochem. Cell Biol..

[93] Sestili P., Alfieri R., Carnicelli D., Martinelli C., Barbieri L., Stirpe F., Bonelli M., Petronini P.G., Brigotti M. (2005). Shiga toxin 1 and ricin inhibit the repair of H2O2-induced DNA single strand breaks in cultured mammalian cells.. DNA Repair.

[94] Li X.-P., Baricevic M., Saidasan H., Tumer N.E. (2007). Ribosome depurination is not sufficient for ricin-mediated cell death in *Saccharomyces cerevisiae*.. Infect. Immun..

[95] Horrix C., Raviv Z., Flescher E., Voss C., Berger M. (2011). Plant ribosome-inactivating proteins type II induce the unfolded protein response in human cancer cells.. Cell Mol. Life Sci..

[96] Alford S.C., Pearson J., Carette A., Ingham R., Howard P. (2009). Alpha-sarcin catalytic activity is not required for cytotoxicity.. BMC Biochem..

[97] Morlon-Guyot J., Helmy M., Lombard-Frasca S., Pignol D., Pieroni G., Beaumelle B. (2003). Identification of the ricin lipase site and implication in cytotoxicity.. J. Biol. Chem..

[98] Nielsen K., Boston R.S. (2001). Ribosome-inactiating proteins: A plant perspective.. Annu. Rev. Plant Physiol. Plant Mol. Biol..

[99] Rüdiger H., Gabius H.-J. (2001). Plant lectins: Occurrence, biochemistry, functions and application. Glycoconj. J..

[100] Van Dammes E.J.M., Hao Q., Chen Y., Barre A., Vandenbussche F., Desmyter S., Rougé P., Peumans W.J. (2001). Ribosome-inactivating proteins: A family of plant proteins that do more than inactivate ribosomes.. Crit. Rev. Plant Sci..

[101] Van Dammes E.J.M., Fouquaert E., Lannoo N., Vandenborre G., Schouppe D., Peumans W.J. (2011). Novel concepts about the role of lectins in the plant cell.. Adv. Exp. Med. Biol..

[102] Strebhardt K., Ullrich A. (2008). Paul Ehrlich’s magic bullet concept: 100 years of progress.. Nat. Rev. Cancer.

[103] Zhou X.-X., Ji F., Zhao J.-L., Cheng L.-F., Xu C.-F. (2010). Anti-cancer activity of anti-p185Her-2 ricin A chain immunotoxin on gastric cancer cells.. J. Gastroenterol. Hepatol..

[104] Spitler L.E., del Rio M., Khentigan A., Wedel N.I., Brophy N.A., Miller L.L., Harkonen W.S., Rosendorf L.L., Lee H.M., Mischak R.P. (1987). Therapy of patients with malignant melanoma using a monoclonal antimelanoma antibody-ricin A chain immunotoxin.. Cancer Res..

[105] Frankel A.E., Woo J.-H., Neville D.M. (2009). Principles of Cancer Biotherapy.

[106] Schindler J., Gajavelli S., Ravandi F., Shen Y., Parekh S., Braunchweig I., Barta S., Ghetie V., Vitetta E., Verma A. (2011). A phase I study of a combination of anti-CD19 and anti-CD22 immunotoxins (combotox) in adult patients with refractory B-lineage acute lymphoblastic leukaemia.. Br. J. Haematol..

[107] Wu A.M., Senter P.D. (2005). Arming antibodies: Prospects and challenges for immunoconjugates.. Nat. Biotechnol..

[108] Vitetta E.S., Thorpe P.E., Uhr J.W. (1993). Immunotoxins: Magic bullets or misguided missiles?. Trends Pharmacol. Sci..

[109] Baluna R., Vitetta E.S. (1997). Vascular leak syndrome: A side effect of immunotherapy.. Immunopharmacology.

[110] Furman R.R., Grossbard M.L., Johnson J.L., Pecora A.L., Cassileth P.A., Jung S.-H., Peterson B.A., Nadler L.M., Freedman A., Bayer R.-L. (2011). A phase III study of anti-B4-blocked ricin as adjuvant therapy post-autologous bone marrow transplant: CALGB 9254.. Leuk. Lymphoma.

[111] Audi J., Belson M., Patel M., Schier J., Osterloh J. (2005). Ricin poisoning: A comprehensive review.. J. Am. Med. Assoc..

[112] Franz D.R., Jaax N.K., Sidell F.R., Takafuji E.T., Franz D.R. (1997). Ricin Toxin. Medical Aspects of Chemical and Biological Warfare.

[113] Zilinskas R.A. (1997). Iraq’s biological weapons.. J. Am. Med. Assoc..

[114] Kirby R. (2004). Ricin toxin: A military history.. CML Army Chem. Rev..

[115] Schieltz D.M., McGrath S.C., McWilliams L.G., Rees J., Bowen M.D., Kools J.J., Dauphin L.A., Gomez-Saladin E., Newton B.N., Stang H.L. (2011). Analysis of active ricin and castor bean proteins in a ricin preparation, castor bean extract, and surface swabs from a public health investigation.. Forensic. Sci. Int..

[116] Moran G.J. (2002). Threats in bioterrorism. II: CDC category B and C agents.. Emerg. Med. Clin. North Am..

[117] Crompton R., Gall D. (1980). Georgi Markov-death in a pellet.. Med. Leg. J..

[118] Despeyroux D., Walker N., Pearce M., Fisher M., McDonnell M., Bailey S.C., Griffiths G.D., Watts P. (2000). Characterization of ricin heterogeneity by electrospray mass spectrometry, capillary electrophoresis, and resonant mirror.. Anal. Biochem..

[119] Thullier P., Griffiths G. (2009). Broad recognition of ricin toxins prepared from a range of Ricinus cultivars using immunochromatographic tests.. Clin. Toxicol. (Phila.).

[120] Ishiguro M., Tomi M., Funatsu G., Funatsu M. (1976). Isolation and chemical properties of a ricin variant from castor bean.. Toxicon.

[121] Leshin J., Danielsen M., Credle J.J., Weeks A., O’Connell K.P., Dretchen K. (2010). Characterization of ricin toxin family members from *Ricinus communis*.. Toxicon.

[122] Chan A.P., Crabtree J., Zhao Q., Lorenzi H., Orvis J., Puiu D., Melake-Berhan A., Jones K.M., Redman J., Chen G. (2010). Draft genome sequence of the oilseed species *Ricinus communis*.. Nat. Biotechnol..

[123] Sehgal P., Khan M., Kumar O., Vijayaraghavan R. (2010). Purification, characterization and toxicity profile of ricin isoforms from castor beans. Food Chem. Toxicol..

[124] Sehgal P., Kumar O., Kameswararao M., Ravindran J., Khan M., Sharma S., Vijayaraghavan R., Prasad G.B.K.S. (2011). Differential toxicity profile of ricin isoforms correlates with their glycosylation levels.. Toxicology.

[125] Fodstad O., Olsnes S., Pihl A. (1976). Toxicity, distribution and elimination of the cancerostatic lectins abrin and ricin after parenteral injection into mice. Br. J. Cancer.

[126] Poli M.A., Roy C., Huebner K.D., Franz D.R., Jaax N.K., Dembek Z.F. (2007). Ricin. Medical Aspects of Biological Warfare.

[127] Wannemacher R.W., Anderson J.B., Salem H., Katz S.A. (2006). Inhalation Ricin: Aerosol Procedures, Animal Toxicology, and Therapy.. Inhalation Toxicology.

[128] Foxwell B.M., Detre S.I., Donovan T.A., Thorpe P.E. (1985). The use of anti-ricin antibodies to protect mice intoxicated with ricin.. Toxicology.

[129] Olsnes S., Pappenheimer A.M., Meren R. (1974). Lectins from *Abrus precatorius* and *Ricinus communis*: II. Hybrid toxins and their interaction with chain-specific antibodies.. J. Immunol..

[130] Olsnes S., Refsnes K., Pihl A. (1974). Mechanism of action of the toxic lectins abrin and ricin.. Nature.

[131] Fodstad O., Johannessen J.V., Schjerven L., Pihl A. (1979). Toxicity of abrin and ricin in mice and dogs.. J. Toxicol. Environ. Health.

[132] He X., McMahon S., Henderson T.D., Griffey S.M., Cheng L.W. (2010). Ricin toxicokinetics and its sensitive detection in mouse sera or feces using immuno-PCR.. PLoS One.

[133] Jang H.Y., Kim J.H. (1993). Isolation and biochemical properties of ricin from *Ricinus communis*.. Korean Biochem. J..

[134] Lin J.-Y., Liu S.-Y. (1986). Studies on the antitumor lectins isolated from the seeds of *Ricinus communis* (castor bean).. Toxicon.

[135] Roy C.J., Hale M., Hartings J.M., Pitt L., Duniho S. (2003). Impact of inhalation exposure modality and particle size on the respiratory deposition of ricin in BALB/c mice.. Inhal. Toxicol..

[136] Derenzini M., Bonetti E., Marionozzi V., Stirpe F. (1976). Toxic effects of ricin: Studies on the pathogenesis of liver lesions.. Virchows Arch. B.

[137] Griffiths G.D., Phillips G.J., Holley J. (2007). Inhalation toxicology of ricin preparations: Animal models, prophylactic and therapeutic approaches to protection. Inhal. Toxicol..

[138] Griffiths G.D., Rice P., Allenby A.C., Bailey S.C., Upshall D.G. (1995). Inhalation toxicology and histopathology of ricin and abrin toxins.. Inhal. Toxicol..

[139] Roy C.J., Reed D.S., Hutt J.A. (2010). Aerobiology and inhalation exposure to biological select agents and toxins.. Vet. Pathol..

[140] Ishiguro M., Mitarai M., Harada H., Sekine I., Nishimori I., Kikutani M. (1983). Biochemical studies on oral toxicity of ricin. I. Ricin administered orally can impair sugar absorption by rat small intestine. Chem. Pharm. Bull. (Tokyo).

[141] Garber E.A.E. (2008). Toxicity and detection of ricin and abrin in beverages.. J. Food Prot..

[142] Cook D.L., David J., Griffiths G.D. (2006). Retrospective identification of ricin in animal tissues following administration by pulmonary and oral routes.. Toxicology.

[143] Tuson R.V. (1864). note on an alkaloid contained in the seeds of the *Ricinus communis*, or castor-oil plant. J. Chem. Soc..

[144] Böttcher B. (1918). Zur Kenntnis des Ricinins.. Ber. Dtsch. Chem. Ges..

[145] Späth E., Koller G. (1923). Die Synthese des Ricinins.. Ber. Dtsch. Chem. Ges..

[146] Späth E., Koller G. (1923). Die Konstitution des Ricinins.. Ber. Dtsch. Chem. Ges..

[147] Soriano-García M., Jiménez M.E., Reyes Vaca R., Toscano R.A. (1989). Structure of ricinine.. Acta Crystallogr. Sec. C.

[148] Waller G.R., Skursky L. (1972). Translocation and metabolism of ricinine in the castor bean plant, *Ricinus communis* L. Plant Physiol..

[149] Mann D.F., Byerrum R.U. (1974). Activation of the *de novo* pathway for pyridine nucleotide biosynthesis prior to ricinine biosynthesis in castor beans.. Plant Physiol..

[150] Ferraz A.C., Angelucci M.E.M., Da Costa M.L., Batista I.R., de Oliveira B.H., da Cunha C. (1999). Pharmacological evaluation of ricinine, a central nervous system stimulant isolated from *Ricinus communis*. Pharmacol. Biochem. Behav..

[151] Ferraz A.C., Pereira L.F., Ribeiro R.L., Wolfman C., Medina J.H., Scorza F.A., Santos N.F., Cavalheiro E.A., da Cunha C. (2000). Ricinine-elicited seizures: A novel chemical model of convulsive seizures.. Pharmacol. Biochem. Behav..

[152] Ferraz A.C., Anselmo-Franci J.A., Perosa S.R., de Castro-Neto E.F., Bellissimo M.I., de Oliveira B.H., Cavalheiro E.A., Naffah-Mazzacoratti M.D.G., da Cunha C. (2002). Amino acid and monoamine alterations in the cerebral cortex and hippocampus of mice submitted to ricinine-induced seizures.. Pharmacol. Biochem. Behav..

[153] De Assis Junior E.M., Fernandes I.M.d.S., Santos C.S., de Mesquita L.X., Pereira R.A., Maracajá P.B., Soto-Blanco B. (2011). Toxicity of castor bean (*Ricinus communis*) pollen to honeybees.. Agric. Ecosyst. Environ..

[154] Upasani S.M., Kotkar H.M., Mendki P.S., Maheshwari V.L. (2003). Partial characterization and insecticidal properties of *Ricinus communis* L. foliage flavonoids.. Pest Manag. Sci..

[155] Bigi M.F.M.A., Torkomian V.L.V., de Groote S.T.C.S., Hebling M.J.A., Bueno O.C., Pagnocca F.C., Fernandes J.B., Vieira P.C., da Silva M.F.G.F. (2004). Activity of *Ricinus communis* (Euphorbiaceae) and ricinine against the leaf-cutting ant *Atta sexdens rubropilosa* (hymenoptera: Formicidae) and the symbiotic fungus Leucoagaricus gongylophorus.. Pest Manag. Sci..

[156] Taylor S., Massiah A., Lomonossoff G., Roberts L.M., Lord J.M., Hartley M. (1994). Correlation between the activities of five ribosome-inactivating proteins in depurination of tobacco ribosomes and inhibition of tobacco mosaic virus infection.. Plant J..

[157] Sitton D., West C.A. (1975). Casbene: An anti-fungal diterpene produced in cell-free extracts of *Ricinus communis* seedlings.. Phytochemistry.

[158] Figley K.D., Elrod R.H. (1928). Endemic asthma due to castor bean dust.. J. Am. Med. Assoc..

[159] Ratner B., Gruehl H.L. (1929). Respiratory anaphylaxis (asthma) and ricin poisoning induced with castor bean dust.. Am. J. Epidemiol..

[160] Alistair E. (1914). Études de la ricine: III. Hypersensibilité a la ricine.. Ann. Inst. Pasteur..

[161] Bashir M.E.H., Hubatsch I., Leinenbach H.P., Zeppezauer M., Panzani R.C., Hussein I.H. (1998). Ric c 1 and Ric c 3, the allergenic 2S albumin storage proteins of *Ricinus communis*: Complete primary structures and phylogenetic relationships. Int. Arch. Allergy Immunol..

[162] Thorpe S.C., Kemeny D.M., Panzani R.C., McGurl B., Lord M. (1988). Allergy to castor bean. II. Identification of the major allergens in castor bean seeds.. J. Allergy Clin. Immunol..

[163] Deus-de-Oliveira N., Felix S.P., Carrielo-Gama C., Fernandes K.V., Damatta R.A., Machado O.L. (2011). Identification of critical amino acids in the IgE epitopes of Ric c 1 and Ric c 3 and the application of glutamic acid as an IgE blocker. PLoS One.

[164] Spies J.R., Coulson E.J. (1965). Antigenic specificity relationships of castor bean meal, pollen, and allergenic fraction, cb-1a, of Ricinus commu. J. Allergy.

[165] Rauber A., Heard J. (1985). Castor bean toxicity re-examined: A new perspective.. Vet. Hum. Toxicol..

[166] Challoner K.R., McCarron M.M. (1990). Castor bean intoxication.. Ann. Emerg. Med..

[167] Balint G.A. (1974). Ricin: The toxic protein of castor oil seeds.. Toxicology.

[168] Reed R.P. (1998). Castor oil seed poisoning: A concern for children.. Med. J. Aust..

[169] Lucas G.N. (2006). Plant poisoning in Sri Lankan children: A hospital based prospective study.. Sri. Lanka. J. Child Health.

[170] Ingle V., Kale V., Talwalkar Y. (1966). Accidental poisoning in children with particular reference to castor beans.. Indian J. Pediatr..

[171] Kinamore P.A., Jaeger R.W., de Castro F.J. (1980). Abrus and ricinus ingestion: Management of three cases.. Clin. Toxicol..

[172] Despott E., Cachia M.J. (2004). A case of accidental ricin poisoning.. Malta Med. J..

[173] Aplin P.J., Eliseo T. (1997). Ingestion of castor oil plant seeds.. Med. J. Aust..

[174] Fine D.R., Shepherd H.A., Griffiths G.D., Green M. (1992). Sub-lethal poisoning by self-injection with ricin.. Med. Sci. Law.

[175] Krenzelok E.P., Mrvos R. (2011). Friends and foes in the plant world: A profile of plant ingestions and fatalities. Clin. Toxicol. (Phila.).

[176] Jaspersen-Schib R., Theus L., Guirguis-Oeschger M., Gossweiler B., Meier-Abt P.J. (1996). Wichtige Pflanzenvergiftungen in der Schweiz 1966−1994: Eine Fallanalyse aus dem schweizerischen toxikologischen Informationszentrum (STIZ).. Schweiz. Med. Wchnschr..

[177] Lim H., Kim H.J., Cho Y.S. (2009). A case of ricin poisoning following ingestion of Korean castor bean.. Emerg. Med. J..

[178] Castex M.R. (1949). Intoxicacion y alergia por la ingestion de semillas de *Ricinus communis*. Prensa Méd. Argent.

[179] Maretić Z. (1980). Otrovanje sjemenkama ricinusa.. Arh. Hig. Rada Toksikol..

[180] Nishiyama T., Oka H., Miyoshi M., Aibiki M., Maekawa S., Shirakawa Y. (2005). Case of accidental ingestion of caster beans: Acute intoxication by ricin.. Chudoku Kenkyu.

[181] Al-Tamimi F.A., Hegazi A.E. (2008). A case of castor bean poisoning.. Sultan Qaboos Univ. Med. J..

[182] Smith S.W., Graber N.M., Johnson R.C., Barr J.R., Hoffman R.S., Nelson L.S. (2009). Multisystem organ failure after large volume injection of castor oil.. Ann. Plast. Surg..

[183] Targosz D., Winnik L., Szkolnicka B. (2002). Suicidal poisoning with castor bean (*Ricinus communis*) extract injected subcutaneously-Case report.. Clin. Toxicol..

[184] Watson W.A., Litovitz T.L., Klein-Schwartz W., Rodgers G.C., Youniss J., Reid N., Rouse W.G., Rembert R.S., Borys D. (2004). 2003 annual report of the American Association of Poison Control Centers toxic exposure surveillance system. Am. J. Emerg. Med..

[185] Coopman V., De Leeuw M., Cordonnier J., Jacobs W. (2009). Suicidal death after injection of a castor bean extract (*Ricinus communis* L.).. Forensic. Sci. Int..

[186] De Paepe P., Gijsenbergh F., Martens F., Piette M., Buylaert W. (2005). Two fatal cases following ricin injection.. Br. J. Clin. Pharmacol..

[187] Passeron T., Mantoux F., Lacour J.P., Roger P.M., Fosse T., Iannelli A., Ortonne J.P. (2004). Infectious and toxic cellulitis due to suicide attempt by subcutaneous injection of ricin.. Br. J. Dermatol..

[188] Milewski L.M., Khan S.A. (2006). An overview of potentially life-threatening poisonous plants in dogs and cats.. J. Vet. Emerg. Crit. Care.

[189] Meldrum W.P. (1900). Poisoning by castor oil seeds.. Br. Med. J..

[190] Bispham W.N. (1903). Report of cases of poisoning by fruit of *Ricinus communis*.. Am. J. Med. Sci..

[191] Burroughs W.J. (1903). Poisonous effects of *Ricinus communis*.. Br. Med. J..

[192] Arnold H.L. (1924). Poisoning from castor bean.. Science.

[193] Lipták P. (1928). Rizinussamen-Vergiftungen.. Arch. Toxicol..

[194] (1935). Abdülkadir-Lütfi; Taeger, Tödliche Vergiftung durch Rizinussamen. Arch. Toxicol..

[195] Möschl H. (1938). Zur Klinik und Pathogenese der Rizinvergiftung.. Wien. Klin. Wchnschr..

[196] Koch L.A., Caplan J. (1942). Castor bean poisoning.. Am. J. Dis. Child.

[197] Abbozzo G. (1953). Klinisch-toxikologische Zusammenstellung der Vergiftungsfälle in Florenz im Triennium 1950-1952.. Arch. Toxicol..

[198] Kaszás T., Papp G. (1960). Ricinussamenvergiftung von Schulkindern.. Arch. Toxikol..

[199] Karolini T., Zarnowska-Cwiertka W. (1965). Case of poisoning with ricinus seeds.. Przegl. Epidemiol..

[200] Krain L.S., Bucher W.H., Heidbreder G.A. (1971). Trends in accidental poisoning in childhood. Los Angeles county experience.. Clin. Pediatr. (Phila.).

[201] Kingma J. (1971). Ricin poisoning caused by chewing a castor bean.. Ned Tijdschr Geneeskd.

[202] Ramakrishnan S., Balasubramanian K., Madhavan M. (1972). Biochemical and pathological studies on castor seed poisoning.. J. Assoc. Physicians India.

[203] Malizia E., Sarcinelli L., Andreucci G. (1977). Ricinus poisoning: A familiar epidemy. Acta Pharmacol. Toxicol. (Copenh.).

[204] Knight B. (1979). Ricin-a potent homicidal poison.. Br. Med. J..

[205] Satpathy R., Das B.B. (1979). Accidental poisoning in childhood.. J. Indian Med. Assoc..

[206] Henry G.W., Schwenk G.R., Bohnert P.A. (1981). Umbrellas and mole beans: A warning about acute ricin poisoning. J. Indiana State Med. Assoc..

[207] Vinther S., Matzen P. (1983). Poisoning with the castor oil plant (*Ricinus communis* L.).. Ugeskr. Laeger..

[208] Romanos A., Toledo F., Vazquez G., Guzman J., Serrano M.L., Velasco Y.M.J.  (1983). Intoxicacion por semillas de *Ricinus communis*. Nota clinica. Rev. Toxicol. (Elche Spain).

[209] Zifroni A. (1985). Castor bean poisoning.. Harefuah.

[210] Painter M.J., Veitch I.H.M., Packer J.M.V. (1985). A science lesson, a castor oil plant seed and a salford schoolboy. Commun. Med..

[211] Wedin G.P., Neal J.S., Everson G.W., Krenzelok E.P. (1986). Castor bean poisoning.. Am. J. Emerg. Med..

[212] Belzunegui O.T., Charles A.B., Hernandez R., Maravi Petri E. (1988). Poisoning by ingestion of castor bean seeds. Apropos of a case.. Med. Clin. (Barc.).

[213] Ravindra F.R., Dulitya F.N. (1990). Poisoning with plants and mushrooms in Sri Lanka: A retrospective hospital based study.. Vet. Hum. Toxicol..

[214] Palatnick W., Tenenbein M. (2000). Hepatotoxicity from castor bean ingestion in a child.. J. Toxicol. Clin. Toxicol..

[215] Hamouda C., Amamou M., Thabet H., Yacoub M. (2000). Plant poisonings from herbal medication admitted to a tunisian toxicologic intensive care unit, 1983-1998. Vet. Hum. Toxicol..

[216] Frohne D., Pfänder H.J. (2004). *Ricinus*  *communis*. Giftpflanzen-Ein Handbuch für Apotheker, Ärzte, Toxikologen und Biologen.

[217] Klain G.J., Jaeger J.J. (1990). Castor Seed Poisoning in Humans: A Review: Technical Report #453.

[218] Papaloucas M., Papaloucas C., Stergioulas A. (2008). Ricin and the assassination of Georgi Markov.. Pak. J. Biol. Sci..

[219] Kopferschmitt J., Flesch F., Lugnier A., Sauder P., Jaeger A., Mantz J.M. (1983). Acute voluntary intoxication by ricin.. Hum. Toxicol..

[220] Garcia F.M., Alvarez A.P., Baneres G.B., Alegre B.V. (1996). Poisoning caused by ricin seeds.. Aten. Primaria.

[221] Alao A.O., Yolles J.C., Armenta W. (1999). Cybersuicide: The internet and suicide.. Am. J. Psychiatry.

[222] Johnson R.C., Lemire S.W., Woolfitt A.R., Ospina M., Preston K.P., Olson C.T., Barr J.R. (2005). Quantification of ricinine in rat and human urine: A biomarker for ricin exposure.. J. Anal. Toxicol..

[223] Krieger-Huber S. (1980). Rizin-Vergiftungen mit tödlichem Ausgang bei Hunden nach Aufnahme des biologischen Naturdüngers “Oscorna Animalin”.. Kleintier Praxis.

[224] Albretsen J.C., Gwaltney-Brant S.M., Khan S.A. (2000). Evaluation of castor bean toxicosis in dogs: 98 cases.. J. Am. Anim. Hosp. Assoc..

[225] Ebbecke M., Hünefeld D., Schaper A., Desl H. (2002). Increasing frequency of serious or fatal poisonings in dogs caused by organic fertilizers during the summer of 2001 in Germany.. Clin. Toxicol..

[226] Soto-Blanco B., Sinhorini I.L., Gorniak S.L., Schumaher-Henrique B. (2002). *Ricinus communis* cake poisoning in a dog.. Vet. Hum. Toxicol..

[227] Cardoso M.J.L., Fernandes H.S., Lima L.S.A., Moutinho F.Q., Sakate M. (2005). Accidental ingestion of *Ricinus communis* in dogs (*Canis familiaris*, L. 1758)-Case report. Vet. Notícias Uberlândia.

[228] Mouser P., Filigenzi M.S., Puschner B., Johnson V., Miller M.A., Hooser S.B. (2007). Fatal ricin toxicosis in a puppy confirmed by liquid chromatography/mass spectrometry when using ricinine as a marker.. J. Vet. Diagn. Invest..

[229] Neika D. (2010). Vergiftung mit Rizinussamen bei einem Hund - Fallbericht.. Kleintiermedizin.

[230] Roels S., Coopman V., Vanhaelen P., Cordonnier J. (2010). Lethal ricin intoxication in two adult dogs: Toxicologic and histopathologic findings.. J. Vet. Diagn. Invest..

[231] Hong I.H., Kwon T.E., Lee S.K., Park J.K., Ki M.R., Park S.I., Jeong K.S. (2011). Fetal death of dogs after the ingestion of a soil conditioner.. Exp. Toxicol. Pathol..

[232] Vigener A. (1874). Untersuchung eines verfälschten Leinmehls.. Arch. Pharm..

[233] (1888). Regensbogen, vergiftung durch Leinsamen bei Pferden. Berl. Tierarztl. Wchnschr..

[234] Geary T. (1950). Castor bean poisoning.. Vet. Rec..

[235] Jensen W.I., Allen J.P. (1981). Naturally occurring and experimentally induced castor bean (*Ricinus communis*) poisoning in ducks.. Avian Dis..

[236] Fernandes W.R., Baccarin R.Y.A., Michima L.E.S. (2002). Equine poisoning by *Ricinus communis*: Case report. Rev. Bras. Saúde Prod. An..

[237] Aslani M.R., Maleki M., Mohri M., Sharifi K., Najjar-Nezhad V., Afshari E. (2007). Castor bean (*Ricinus communis*) toxicosis in a sheep flock.. Toxicon.

[238] Miessner H. (1909). Ueber die Giftigkeit der Rizinussamen. Mitt. des Kaiser Wilhelm-Instituts für Landwirtschaft in Bromberg.

[239] (2009). Commission Directive 2009/141/EC of 23.11.2009. Off J Eur Union L.

[240] Düngemittelverordnung -DüMv-, Attachment 2, Nr. 7.1.5.. http://www.gesetze-im-internet.de/bundesrecht/d_mv_2008/gesamt.pdf.

[241] Koja N., Shibata T., Mochida K. (1980). Enzyme-linked immunoassay of ricin.. Toxicon.

[242] Poli M.A., Rivera V.R., Hewetson J.F., Merrill G.A. (1994). Detection of ricin by colorimetric and chemiluminescence ELISA.. Toxicon.

[243] Frankel A.E., Burbage C., Fu T., Tagge E., Chandler J., Willingham M. (1996). Characterization of a ricin fusion toxin targeted to the interleukin-2 receptor.. Protein Eng..

[244] Griffiths G.D., Newman H., Gee D.J. (1986). Identification and quantification of ricin toxin in animal tissues using ELISA.. J. Forensic. Sci. Soc..

[245] Leith A.G., Griffiths G.D., Green M.A. (1988). Quantification of ricin toxin using a highly sensitive avidin/biotin enzyme-linked immunosorbent assay.. J. Forensic. Sci. Soc..

[246] Pauly D., Kirchner S., Störmann B., Schreiber T., Kaulfuss S., Schade R., Zbinden R., Avondet M.A., Dorner M.B., Dorner B.G. (2009). Simultaneous quantification of five bacterial and plant toxins from complex matrices using a multiplexed fluorescent magnetic suspension assay.. Analyst.

[247] Rubina A.Y., Dyukova V.I., Dementieva E.I., Stomakhin A.A., Nesmeyanov V.A., Grishin E.V., Zasedatelev A.S. (2005). Quantitative immunoassay of biotoxins on hydrogel-based protein microchips.. Anal. Biochem..

[248] Guglielmo-Viret V., Splettstoesser W., Thullier P. (2007). An immunochromatographic test for the diagnosis of ricin inhalational poisoning.. Clin. Toxicol..

[249] Zhuang J., Cheng T., Gao L., Luo Y., Ren Q., Lu D., Tang F., Ren X., Yang D., Feng J. (2010). Silica coating magnetic nanoparticle-based silver enhancement immunoassay for rapid electrical detection of ricin toxin.. Toxicon.

[250] Lang L., Wang Y., Wang C., Zhao Y., Jia P., Fu F. (2009). Determination of ricin by double antibody sandwich enzyme-linked immunosorbent assay in different samples.. J. Int. Pharm. Res..

[251] Men J., Lang L., Wang C., Wu J., Zhao Y., Jia P.Y., Wei W., Wang Y. (2010). Detection of residual toxin in tissues of ricin-poisoned mice by sandwich enzyme-linked immunosorbent assay and immunoprecipitation.. Anal. Biochem..

[252] Shyu H.F., Chiao D.J., Liu H.W., Tang S.S. (2002). Monoclonal antibody-based enzyme immunoassay for detection of ricin.. Hybrid. Hybrid..

[253] Garber E.A., O’Brien T.W. (2008). Detection of ricin in food using electrochemiluminescence-based technology.. J. AOAC Int..

[254] He X., McMahon S., McKeon T.A., Brandon D.L. (2010). Development of a novel immuno-PCR assay for detection of ricin in ground beef, liquid chicken egg, and mil. J. Food Prot..

[255] Zhang H., Zhao Q., Li X.-F., Le X.C. (2007). Ultrasensitive assays for proteins.. Analyst.

[256] Lubelli C., Chatgilialoglu A., Bolognesi A., Strocchi P., Colombatti M., Stirpe F. (2006). Detection of ricin and other ribosome-inactivating proteins by an immuno-polymerase chain reaction assay.. Anal. Biochem..

[257] Narang U., Anderson G.P., Ligler F.S., Burans J. (1997). Fiber optic-based biosensor for ricin.. Biosens. Bioelectron..

[258] Shyu R.-H., Shyu H.-F., Liu H.-W., Tang S.-S. (2002). Colloidal gold-based immunochromatographic assay for detection of ricin.. Toxicon.

[259] Weber M., Schulz H. (2011). Immunological detection of ricin and castor seeds in beverages, food and consumer products. Toxichem. Krimtech..

[260] Dayan-Kenigsberg J., Bertocchi A., Garber E.A. (2008). Rapid detection of ricin in cosmetics and elimination of artifacts associated with wheat lectin.. J. Immunol. Methods.

[261] Ding S., Gao C., Gu L.-Q. (2009). Capturing single molecules of immunoglobulin and ricin with an aptamer-encoded glass nanopore.. Anal. Chem..

[262] Kirby R., Cho E.J., Gehrke B., Bayer T., Park Y.S., Neikirk D.P., McDevitt J.T., Ellington A.D. (2004). Aptamer-based sensor arrays for the detection and quantitation of proteins.. Anal. Chem..

[263] Tang J., Xie J., Shao N., Yan Y. (2006). The DNA aptamers that specifically recognize ricin toxin are selected by two *in vitro* selection methods.. Electrophoresis.

[264] Haes A.J., Giordano B.C., Collins G.E. (2006). Aptamer-based detection and quantitative analysis of ricin using affinity probe capillary electrophoresis.. Anal. Chem..

[265] Förster C., Oberthuer D., Gao J., Eichert A., Quast F.G., Betzel C., Nitsche A., Erdmann V.A., Furste J.P. (2009). Crystallization and preliminary X-ray diffraction data of an LNA 7-mer duplex derived from a ricin aptamer.. Acta Crystallogr. Sec. F.

[266] Hesselberth J.R., Miller D., Robertus J., Ellington A.D. (2000). In vitro selection of RNA molecules that inhibit the activity of ricin A-chain.. J. Biol. Chem..

[267] Fan S., Wu F., Martiniuk F., Hale M.L., Ellington A.D., Tchou-Wong K.M. (2008). Protective effects of anti-ricin A-chain RNA aptamer against ricin toxicity.. World J. Gastroenterol..

[268] Lamont E.A., He L., Warriner K., Labuza T.P., Sreevatsan S. (2011). A single DNA aptamer functions as a biosensor for ricin.. Analyst.

[269] Jayasena S.D. (1999). Aptamers: An emerging class of molecules that rival antibodies in diagnostics.. Clin. Chem..

[270] stin A., Bergström T., Fredriksson S.A., Nilsson C. (2007). Solvent-assisted trypsin digestion of ricin for forensic identification by LC-ESI MS/MS.. Anal. Chem..

[271] Fredriksson S.A., Hulst A.G., Artursson E., de Jong A.L., Nilsson C., van Baar B.L. (2005). Forensic identification of neat ricin and of ricin from crude castor bean extracts by mass spectrometry.. Anal. Chem..

[272] Brinkworth C.S., Pigott E.J., Bourne D.J. (2009). Detection of intact ricin in crude and purified extracts from castor beans using matrix-assisted laser desorption ionization mass spectrometry.. Anal. Chem..

[273] Brinkworth C.S. (2010). Identification of ricin in crude and purified extracts from castor beans using on-target tryptic digestion and MALDI mass spectrometry.. Anal. Chem..

[274] Norrgran J., Williams T.L., Woolfitt A.R., Solano M.I., Pirkle J.L., Barr J.R. (2009). Optimization of digestion parameters for protein quantification.. Anal. Biochem..

[275] Kull S., Pauly D., Störmann B., Kirchner S., Stämmler M., Dorner M.B., Lasch P., Naumann D., Dorner B.G. (2010). Multiplex detection of microbial and plant toxins by immunoaffinity enrichment and matrix-assisted laser desorption/ionization mass spectrometry.. Anal. Chem..

[276] Sehgal P., Rao M.K., Kumar O., Vijayaraghavan R. (2010). Characterization of native and denatured ricin using MALDI-ToF/MS.. Cell Mol. Biol. (Noisy-le-grand).

[277] Kalb S.R., Barr J.R. (2009). Mass spectrometric detection of ricin and its activity in food and clinical samples.. Anal. Chem..

[278] McGrath S.C., Schieltz D.M., McWilliams L.G., Pirkle J.L., Barr J.R. (2011). Detection and quantification of ricin in beverages using isotope dilution tandem mass spectrometry.. Anal. Chem..

[279] Duriez E., Fenaille F., Tabet J.C., Lamourette P., Hilaire D., Becher F., Ezan E. (2008). Detection of ricin in complex samples by immunocapture and matrix-assisted laser desorption/ionization time-of-flight mass spectrometry.. J. Proteome Res..

[280] Kumar O., Pradhan S., Sehgal P., Singh Y., Vijayaraghavan R. (2010). Denatured ricin can be detected as native ricin by immunological methods, but nontoxic *in vivo*. J. Forensic. Sci..

[281] Lumor S.E., Hutt A., Ronningen I., Diez-Gonzalez F., Labuza T.P. (2011). Validation of immunodetection (ELISA) of ricin using a biological activity assay.. J. Food Sci..

[282] Jackson L.S., Zhang Z., Tolleson W.H. (2010). Thermal stability of ricin in orange and apple juices.. J. Food Sci..

[283] Jackson L.S., Tolleson W.H., Chirtel S.J. (2006). Thermal inactivation of ricin using infant formula as a food matrix.. J. Agric. Food Chem..

[284] Ishiguro M., Takahashi T., Funatsu G., Hayashi K., Funatsu M. (1964). Biochemical studies on ricin: I. Purification of ricin.. J. Biochem..

[285] Brzezinski J.L., Craft D.L. (2007). Evaluation of an *in vitro* bioassay for the detection of purified ricin and castor bean in beverages and liquid food matrices.. J. Food Prot..

[286] Cole K.D., Gaigalas A., Almeida J.L. (2008). Process monitoring the inactivation of ricin and model proteins by disinfectants using fluorescence and biological activity.. Biotechnol. Prog..

[287] Lin J.-Y., Liu K., Chen C.-C., Tung T.-C. (1971). Effect of crystalline ricin on the biosynthesis of protein, RNA, and DNA in experimental tumor cell. Cancer Res..

[288] Olsnes S. (1972). Toxic proteins inhibiting protein synthesis.. Naturwissenschaften.

[289] Olsnes S., Pihl A. (1972). Ricin-A potent inhibitor of protein synthesis.. FEBS Lett..

[290] He X., Lu S., Cheng L.W., Rasooly R., Carter J.M. (2008). Effect of food matrices on the biological activity of ricin.. J. Food Prot..

[291] Hale M.L. (2001). Microtiter-based assay for evaluating the biological activity of ribosome-inactivating proteins.. Pharmacol. Toxicol..

[292] Heisler I., Keller J., Tauber R., Sutherland M., Fuchs H. (2002). A colorimetric assay for the quantitation of free adenine applied to determine the enzymatic activity of ribosome-inactivating proteins.. Anal. Biochem..

[293] Zamboni M., Brigotti M., Rambelli F., Montanaro L., Sperti S. (1989). High-pressure-liquid-chromatographic and fluorimetric methods for the determination of adenine released from ribosomes by ricin and gelonin.. Biochem. J..

[294] Roday S., Sturm M.B., Blakaj D., Schramm V.L. (2008). Detection of an abasic site in RNA with stem-loop DNA beacons: Application to an activity assay for ricin toxin A-chain.. J. Biochem. Biophys. Methods.

[295] Hines H.B., Brueggemann E.E., Hale M.L. (2004). High-performance liquid chromatography-mass selective detection assay for adenine released from a synthetic RNA substrate by ricin A chain.. Anal. Biochem..

[296] Keener W.K., Rivera V.R., Young C.C., Poli M.A. (2006). An activity-dependent assay for ricin and related RNA *N-*glycosidases based on electrochemiluminescence.. Anal. Biochem..

[297] Pierce M., Kahn J.N., Chiou J., Tumer N.E. (2011). Development of a quantitative RT-PCR assay to examine the kinetics of ribosome depurination by ribosome inactivating proteins using saccharomyces cerevisiae as a model.. RNA.

[298] Becher F., Duriez E., Volland H., Tabet J.C., Ezan E. (2007). Detection of functional ricin by immunoaffinity and liquid chromatography-tandem mass spectrometry.. Anal. Chem..

[299] Ishiguro M., Tanabe S., Matori Y., Sakakibara R. (1992). Biochemical studies on oral toxicity of ricin. IV. A fate of orally administered ricin in rats.. J. Pharmacobiodyn..

[300] Bingen A., Creppy E.E., Gut J.P., Dirheimer G., Kirn A. (1987). The Kupffer cell is the first target in ricin-induced hepatitis.. J. Submicrosc. Cytol..

[301] Zenilman M.E., Fiani M., Stahl P.D., Brunt E.M., Flye M.W. (1989). Selective depletion of Kupffer cells in mice by intact ricin.. Transplantation.

[302] Zenilman M.E., Fiani M., Stahl P.D., Brunt E.M., Flye M.W. (1988). Use of ricin A-chain to selectively deplete Kupffer cells.. J. Surg. Res..

[303] Magnusson S., Berg T. (1993). Endocytosis of ricin by rat liver cells *in vivo* and *in vitro* is mainly mediated by mannose receptors on sinusoidal endothelial cells.. Biochem. J..

[304] Skilleter D.N., Paine A.J., Stirpe F. (1981). A comparison of the accumulation of ricin by hepatic parenchymal and non-parenchymal cells and its inhibition of protein synthesis.. Biochim. Biophys. Acta.

[305] McGreal E.P., Miller J.L., Gordon S. (2005). Ligand recognition by antigen-presenting cell C-type lectin receptors.. Curr. Opin. Immunol..

[306] Bilzer M., Roggel F., Gerbes A.L. (2006). Role of Kupffer cells in host defense and liver disease.. Liver Int..

[307] Robinson T., Fowell E. (1959). A chromatographic analysis for ricinine.. Nature.

[308] Hinkson J., Elliger C., Fuller G. (1972). The effect of ammoniation upon ricinine in castor meal.. J. Am. Oil Chem. Soc..

[309] Olaifa J.I., Matsumura F., Zeevaart J.A.D., Mullin C.A., Charalambous P. (1991). Lethal amounts of ricinine in green peach aphids (*Myzus persicae*) (suzler) fed on castor bean plants.. Plant Sci..

[310] Darby S.M., Miller M.L., Allen R.O. (2001). Forensic determination of ricin and the alkaloid marker ricinine from castor bean extracts.. J. Forensic. Sci..

[311] Melchert H.U., Pabel E. (2004). Reliable identification and quantification of trichothecenes and other mycotoxins by electron impact and chemical ionization-gas chromatography-mass spectrometry, using an ion-trap system in the multiple mass spectrometry mode. Candidate reference method for complex matrices.. J. Chromatogr. A.

[312] Wang Z., Li D., Zhou Z., Li B., Yang W. (2009). A simple method for screening and quantification of ricinine in feed with HPLC and LC-MS.. J. Chromatogr. Sci..

[313] Pruet J.M., Jasheway K.R., Manzano L.A., Bai Y., Anslyn E.V., Robertus J.D. (2011). 7-substituted pterins provide a new direction for ricin A chain inhibitors.. Eur. J. Med. Chem..

[314] Muldoon D.F., Stohs S.J. (1994). Modulation of ricin toxicity in mice by biologically active substances.. J. Appl. Toxicol..

[315] Poli M.A., Rivera V.R., Pitt M.L., Vogel P. (1996). Aerosolized specific antibody protects mice from lung injury associated with aerosolized ricin exposure.. Toxicon.

[316] Pratt T.S., Pincus S.H., Hale M.L., Moreira A.L., Roy C.J., Tchou-Wong K.-M. (2007). Oropharyngeal aspiration of ricin as a lung challenge model for evaluation of the therapeutic index of antibodies against ricin a-chain for post-exposure treatment.. Exp. Lung Res..

[317] Ehrlich P. (1891). Experimentelle Untersuchungen über Immunität.. I. Ueber Ricin. Dtsch. Med. Wchnschr..

[318] Beyer N.H., Kogutowska E., Hansen J.J., Engelhart Illigen K.E., Heegaard N.H. (2009). A mouse model for ricin poisoning and for evaluating protective effects of antiricin antibodies.. Clin. Toxicol. (Phila.).

[319] Pauly D., Dorner M., Zhang X., Hlinak A., Dorner B., Schade R. (2009). Monitoring of laying capacity, immunoglobulin Y concentration, and antibody titer development in chickens immunized with ricin and botulinum toxins over a two-year perio. Poult. Sci..

[320] Hewetson J.F., Rivera V.R., Creasia D.A., Lemley P.V., Rippy M.K., Poli M.A. (1993). Protection of mice from inhaled ricin by vaccination with ricin or by passive treatment with heterologous antibody.. Vaccine.

[321] Stéphanoff M.A. (1896). tudes sur la ricine et l’antiricine.. Ann. Inst. Pasteur..

[322] Houston L.L. (1982). Protection of mice from ricin poisoning by treatment with antibodies directed against ricin.. Clin. Toxicol..

[323] Lemley P.V., Thalley B.S., Stafford D.C. (1995). Prophylactic and therapeutic efficacy of an avian antitoxin in ricin intoxication.. Ther. Immunol..

[324] Godal A., Fodstad Ø., Pihl A. (1983). Antibody formation against the cytotoxic proteins abrin and ricin in humans and mice.. Int. J. Cancer.

[325] Wang Y., Guo L., Zhao K., Chen J., Feng J., Sun Y., Li Y., Shen B. (2007). Novel chimeric anti-ricin antibody C4C13 with neutralizing activity against ricin toxicity.. Biotechnol. Lett..

[326] Pelat T., Hust M., Hale M., Lefranc M.P., Dubel S., Thullier P. (2009). Isolation of a human-like antibody fragment (scFv) that neutralizes ricin biological activity.. BMC Biotechnol..

[327] Pang Y.P., Park J.G., Wang S., Vummenthala A., Mishra R.K., McLaughlin J.E., Di R., Kahn J.N., Tumer N.E., Janosi L. (2011). Small-molecule inhibitor leads of ribosome-inactivating proteins developed using the doorstop approach. PLoS One.

[328] Bai Y., Monzingo A.F., Robertus J.D. (2009). The X-ray structure of ricin A chain with a novel inhibitor.. Arch. Biochem. Biophys..

[329] Hartley P.G., Alderton M.R., Dawson R.M., Wells D. (2007). Ricin antitoxins based on lyotropic mesophases containing galactose amphiphiles.. Bioconj. Chem..

[330] Dawson R.M., Alderton M.R., Wells D., Hartley P.G. (2006). Monovalent and polyvalent carbohydrate inhibitors of ricin binding to a model of the cell-surface receptor.. J. Appl. Toxicol..

[331] Stechmann B., Bai S.K., Gobbo E., Lopez R., Merer G., Pinchard S., Panigai L., Tenza D., Raposo G., Beaumelle B. (2010). Inhibition of retrograde transport protects mice from lethal ricin challenge.. Cell.

[332] Smallshaw J.E., Richardson J.A., Pincus S., Schindler J., Vitetta E.S. (2005). Preclinical toxicity and efficacy testing of RiVax, a recombinant protein vaccine against ricin. Vaccine.

[333] Smallshaw J.E., Richardson J.A., Vitetta E.S. (2007). RiVax, a recombinant ricin subunit vaccine, protects mice against ricin delivered by gavage or aeroso. Vaccine.

[334] Smallshaw J.E., Vitetta E.S. (2010). A lyophilized formulation of RiVax, a recombinant ricin subunit vaccine, retains immunogenicit. Vaccine.

[335] Vitetta E.S., Smallshaw J.E., Coleman E., Jafri H., Foster C., Munford R., Schindler J. (2006). A pilot clinical trial of a recombinant ricin vaccine in normal humans.. Proc. Natl. Acad. Sci. USA.

